# CRISPR technology towards genome editing of the perennial and semi-perennial crops citrus, coffee and sugarcane

**DOI:** 10.3389/fpls.2023.1331258

**Published:** 2024-01-08

**Authors:** Guilherme Souza Prado, Dhiôvanna Corrêia Rocha, Lucas Nascimento dos Santos, Danyel Fernandes Contiliani, Paula Macedo Nobile, Juliana Camargo Martinati-Schenk, Lilian Padilha, Mirian Perez Maluf, Greice Lubini, Tiago Campos Pereira, Claudia Barros Monteiro-Vitorello, Silvana Creste, Raquel Luciana Boscariol-Camargo, Marco Aurélio Takita, Mariângela Cristofani-Yaly, Alessandra Alves de Souza

**Affiliations:** ^1^ Citrus Research Center “Sylvio Moreira” – Agronomic Institute (IAC), Cordeirópolis, Brazil; ^2^ Institute of Biology, State University of Campinas (Unicamp), Campinas, Brazil; ^3^ Sugarcane Research Center – Agronomic Institute (IAC), Ribeirão Preto, Brazil; ^4^ Ribeirão Preto Medical School, University of São Paulo (USP), Ribeirão Preto, Brazil; ^5^ Coffee Center of the Agronomic Institute of Campinas (IAC), Campinas, Brazil; ^6^ Embrapa Coffee, Brazilian Agricultural Research Corporation, Brasília, Federal District, Brazil; ^7^ Department of Biology, Faculty of Philosophy, Sciences and Letters at Ribeirão Preto, University of São Paulo (USP), Ribeirão Preto, Brazil; ^8^ Department of Genetics, Luiz de Queiroz College of Agriculture (ESALQ), University of São Paulo (USP), Piracicaba, Brazil

**Keywords:** edited plants, genome editing tools, perennial crops, regulatory scenario, biotechnology

## Abstract

Gene editing technologies have opened up the possibility of manipulating the genome of any organism in a predicted way. CRISPR technology is the most used genome editing tool and, in agriculture, it has allowed the expansion of possibilities in plant biotechnology, such as gene knockout or knock-in, transcriptional regulation, epigenetic modification, base editing, RNA editing, prime editing, and nucleic acid probing or detection. This technology mostly depends on *in vitro* tissue culture and genetic transformation/transfection protocols, which sometimes become the major challenges for its application in different crops. Agrobacterium-mediated transformation, biolistics, plasmid or RNP (ribonucleoprotein) transfection of protoplasts are some of the commonly used CRISPR delivery methods, but they depend on the genotype and target gene for efficient editing. The choice of the CRISPR system (Cas9, Cas12), CRISPR mechanism (plasmid or RNP) and transfection technique (Agrobacterium spp., PEG solution, lipofection) directly impacts the transformation efficiency and/or editing rate. Besides, CRISPR/Cas technology has made countries rethink regulatory frameworks concerning genetically modified organisms and flexibilize regulatory obstacles for edited plants. Here we present an overview of the state-of-the-art of CRISPR technology applied to three important crops worldwide (citrus, coffee and sugarcane), considering the biological, methodological, and regulatory aspects of its application. In addition, we provide perspectives on recently developed CRISPR tools and promising applications for each of these crops, thus highlighting the usefulness of gene editing to develop novel cultivars.

## 1 Introduction

Since the advent of genetic engineering with the creation of the first recombinant DNA molecules in the 1970s (Jackson et al., 1972; Cohen et al., 1973), recombinant DNA technology has evolved to reach a new phase with the field of synthetic biology (Benner and Sismour, 2005). Although synthetic biology has its roots traced to a landmark publication in 1961 (Jacob and Monod, 1961), it significantly matured and scaled-up in the period from 2008 to 2013 (Cameron et al., 2014), in which novel and remarkable molecular cloning techniques could be used to generate complex gene constructs (Engler et al., 2008; Gibson et al., 2009; Engler et al., 2014), thus paving the way for wide-ranging applications through genome engineering tools.

These tools have been incorporated into plant biotechnology over the last decades, finally enabling the advent of New Breeding Techniques (NBTs) as the future of plant genetic manipulation (Ricroch et al., 2022). Hence, meganucleases (Chilton and Que, 2003), ZFNs (Zinc-finger nucleases) (Wright et al., 2005) and TALENs (Transcription activator-like effector nucleases) (Cermak et al., 2011; Mahfouz et al., 2011) had their use consecutively inaugurated in plants until the most recent generation of gene editor tools, based on CRISPR/Cas (Clustered regularly interspaced short palindromic repeats/CRISPR-associated protein) systems. CRISPR technology (Jinek et al., 2012) is the most sophisticated and practical genome editing approach and was firstly applied to the development of edited plants ten years ago (Feng et al., 2013; Shan et al., 2013).

CRISPR/Cas systems remain the most modern editing tool to date, and it has been increasingly improved or adapted (Cardi et al., 2023), being boosted even by nanotechnology-based delivery systems (Demirer et al., 2021). CRISPR systems in biotechnology are derived from the natural adaptive ‘immune system’ of bacteria and archaea, based on RNA-guided endonucleases that bind and cleave foreign nucleic acids. In nature, these nucleases called Cas effectors are guided to target genome sites as a complex by coupling to a pair of CRISPR RNAs (crRNAs) and *trans*-activating crRNAs (tracrRNAs), thus cleaving the target site located next to a PAM (protospacer adjacent motif) sequence after forming an RNA/DNA heteroduplex between crRNA and host DNA strand (Jinek et al., 2012; Anzalone et al., 2020). This molecular system was engineered for biotech purposes by fusing both crRNA and tracrRNA into a single guide RNA (sgRNA), and the editing mechanism is based on the *indels* (insertions/deletions) or mutagenesis triggered by repair mechanisms occurring in the host cell after target cleavage by double-strand breaks (DSBs), which are mostly mediated by non-homologous end-joining (NHEJ), but also homology-directed repair (HDR) (Jinek et al., 2012).

**Figure 1:**
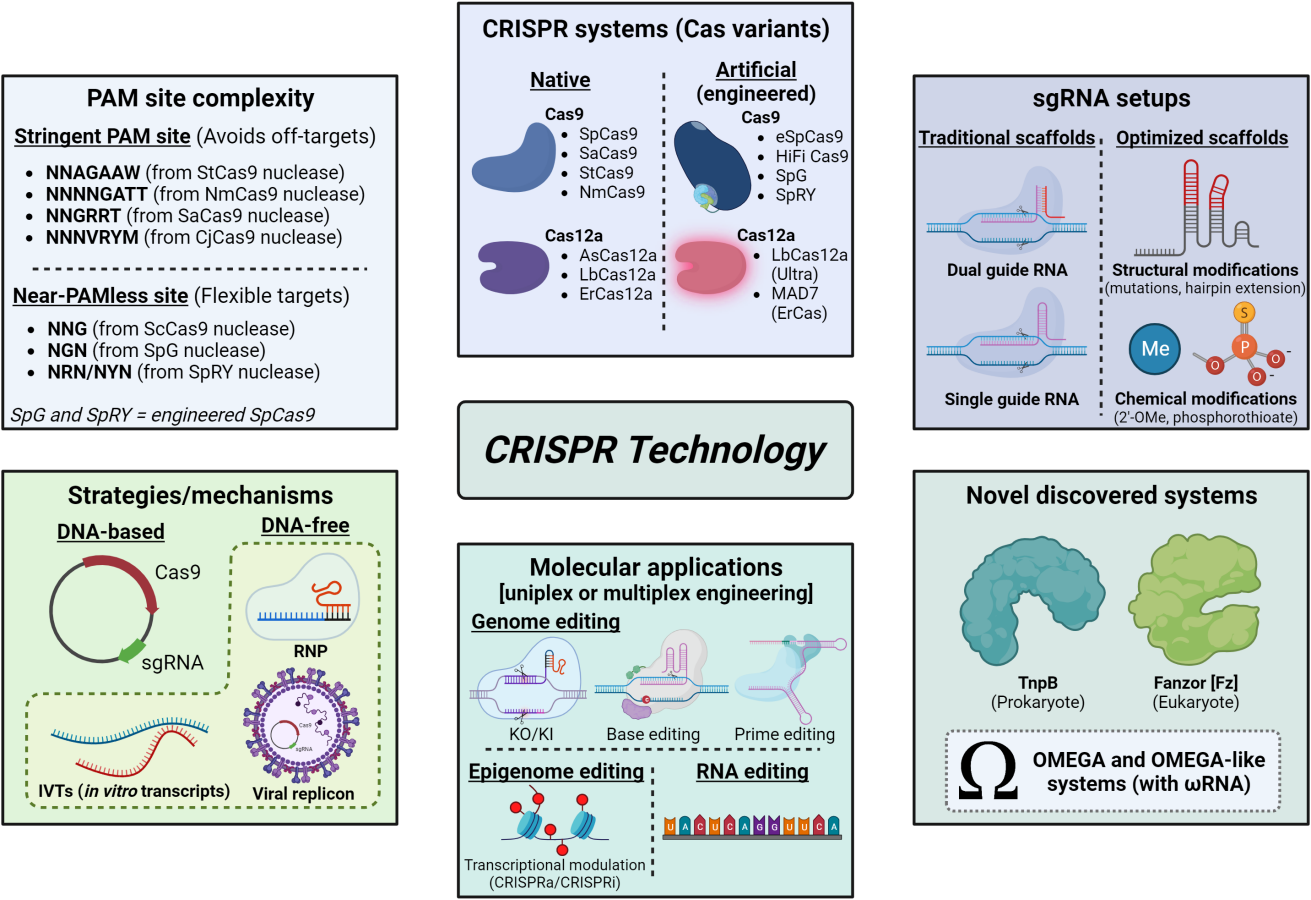
Panorama of CRISPR/Cas and CRISPR-like systems, strategies, applications and optimizations. Methodologies of CRISPR technology vary according to many parameters, as indicated in each box: PAM site complexity, which can be very stringent or even near-PAMless, and then is related to the frequency of on or off-targets in the host genome; the CRISPR system chosen, based on different Cas variants (which can be native or artificial) used to perform gene editing; sgRNA setups, which make use of traditional or optimized scaffolds (in this last case, sgRNA can be structurally and/or chemically modified in order to increase stability and/or gene editing efficiency); strategies/mechanisms adopted for the delivery of CRISPR components, which can be plasmid-based or DNA-free (RNPs, IVTs or viral replicons); and applications (e.g., genome editing, epigenome editing for transcriptional modulation, RNA editing, nucleic acid probing). Moreover, recently discovered and characterized CRISPR-like systems (OMEGA) may be promising for use in agriculture, such as TnpB from prokaryotes and Fanzor (Fz) from eukaryotes, both based on ωRNA scaffolds, showed in the box “Novel discovered systems”. Image created with BioRender.com.

Currently, CRISPR/Cas systems are known as ‘genetic scissors’ and make use of a high diversity of nucleases (**Figure 1**), either naturally occurring from different bacterial species or artificial/engineered variants, each one with their respective PAM sequence (Pickar-Oliver and Gersbach, 2019; Anzalone et al., 2020). CRISPR/SpCas9 (from *Streptococcus pyogenes*) is by far the most used one in any living host (Anzalone et al., 2020; Cardi et al., 2023), including plants (Abdul Aziz et al., 2022), but CRISPR/Cas12a (formerly Cpf1) editing systems have been discovered, optimized and applied to plants as well (Bernabé-Orts et al., 2019) with many advantages and very promising applications. Likewise, other potential successors or novel CRISPR-like systems (**Figure 1**) with very similar activity and mechanisms have been discovered in the last years and have also great potential for plant biotechnology applications, such as the new class of prokaryote transposon-encoded TnpB RNA-guided system named OMEGA (Obligate mobile element-guided activity) (Altae-Tran et al., 2021) and the newly discovered Fanzor (Fz) OMEGA-like programmable system existing in eukaryotes (Saito et al., 2023), both of them phylogenetically related to Cas12 proteins.

Regardless of the system of choice, CRISPR technology can be used for many strategies, such as gene knockout or knock-in, transcriptional regulation, epigenetic modification, base editing, RNA editing, prime editing and nucleic acid probing or detection (**Figure 1**) (Pickar-Oliver and Gersbach, 2019; Anzalone et al., 2020). However, experimental parameters for the CRISPR strategy depend on the intended application and desired organism trait: (a) cell type or explant used, (b) effector nuclease, (c) CRISPR mechanism (plasmid or DNA-free) (d) delivery method and (e) transfection/transformation technique (Anzalone et al., 2020). In general, strategies based on DNA-free mechanisms are the most suitable when the edited plant is wished to reach the market. This is because a transgene-free product may turn the licensing and market approval processes more feasible if considered as non-GMO in a case-by-case analysis, thus making DNA-free editing the most desirable scenario (Kocsisova and Coneva, 2023). In this context, we can classify CRISPR/Cas systems according to the SDN (site-directed nuclease) approach: SDN-1 refers to the introduction of simple random mutations (i.e., substitutions, insertions or deletions) into the host genome through NHEJ mechanism; SDN-2 denotes the replacement of small segments or even single nitrogen bases at the cleaved target site through template recombination carrying desired mutations by using HDR mechanism; and SDN-3 stands for the insertion of at least one large fragment/genetic element (e.g., promoter, CDS and/or terminator) at the target site also using HDR and, differently to what occurs for SDN-1 and SDN-2, SDN-3 is based on the introduction of exogenous sequences in the host genome (Molinari et al., 2021; Rostoks, 2021; Abdul Aziz et al., 2022; Cardi et al., 2023).

In this review, we first present an overview on the state-of-the-art in CRISPR technology applied to agriculture, mainly involving regulatory aspects of plant gene editing around the world, and proceed focusing on three of the most economically and industrially important crops worldwide (citrus, coffee and sugarcane). At this point, we emphasize the aimed traits with CRISPR-mediated crop breeding by giving many research examples reported in the literature. Moreover, we point out the methods for genetic transformation of each crop and correlate them to the main techniques and strategies already employed for their genome editing, without neglecting the main drawbacks and bottlenecks usually faced by researchers for this purpose. We also provide perspectives on recently developed CRISPR tools and promising applications for each crop and what the novel variants and optimizations of CRISPR technology could supply. Finally, we discuss the development of next-generation edited plants based on what is most urgent and feasible for improving these crops.

## 2 CRISPR technology in agriculture

Undoubtedly, gene editing approaches bring numerous potential applications to agriculture. To our knowledge, the first use of gene editing via CRISPR in crop species were published in 2013 (Feng et al., 2013; Shan et al., 2013), just a few months after the inception of the technology in bacteria and animal cells. Thus, fortuitously, the power of this genetic tool was rapidly harnessed and transferred to plant species as a successful example of technical democratization, which is a key feature of CRISPR.

Since then, several genetic modifications via CRISPR have been performed in various economically important plant species (**Table 1**). Among them, we may cite soybean (*Glycine max*) (Jacobs et al., 2015), cassava (*Manihot esculenta*) (Odipio et al., 2017), banana (*Musa* spp.) (Kaur et al., 2018), coffee (*Coffea canephora*) (Casarin et al., 2022), grapevine (*Vitis vinifera*) (Malnoy et al., 2016), rice (*Oryza sativa*) (Lowder et al., 2015), wheat (*Triticum aestivum*) (Upadhyay et al., 2013) and sweet orange (*Citrus sinensis*) (Wang et al., 2014). And the list grows quickly, reaching species once intractable by conventional genetic methodologies. Thus, the possibility of precisely manipulating the genome of any plant in a predicted way, based on limited DNA information (a guide sequence of 20 nucleotides, usually), launches new hope for scientists working with recalcitrant species.

**Table 1:** List of CRISPR-based genome editing studies in crops.

Species	Target gene	Trait	CRISPR delivery method	Reference
*Glycine max*	*GFP* transgene	Loss of fluorescence	*Agrobacterium*-mediated transformation	Jacobs et al., 2015
*Manihot esculenta*	*PDS*	Albino phenotype		Odipio et al., 2017
*Musa* spp.	*PDS*	Albino phenotype		Kaur et al., 2018
*Coffea canephora*	*PDS*	Albino phenotype		Casarin et al., 2022
*Oryza sativa*	*YSA*	Albino phenotype		Lowder et al., 2015
*Triticum aestivum*	*PDS*	Albino phenotype		Upadhyay et al., 2013
*Hordeum vulgare*	*LFY*	Abnormal flowering		Selva et al., 2021
*Lactuca sativa*	*GGP2*	Increased oxidation stress tolerance and ascorbate content		Zhang et al., 2018
*Brassica napus*	*FAD1* and *FAD2*	Increased oleic acid content		Shi et al., 2022
*Solanum lycopersicum*	*PELO* and *MLO1*	Resistance to powdery mildew		Pramanik et al., 2021
*Arachis* sp.	*FAD2*	Increased oleic acid content		Neelakandan et al., 2022
*Fragaria vesca*	*ARF8*	Faster plant growth		Zhou et al., 2018
*Cucumis melo*	*PDS*	Albino phenotype		Hooghvorst et al., 2019
*Cucurbita moschata*	*HKT1;1*	Salt stress sensitivity		Geng et al., 2022
*Cucumis sativus*	*ERECTA*	Shorter internodes		Xin et al., 2022
*Populus davidiana x P. bolleana*	*PDS*	Albino phenotype		Wang et al., 2020
*Dioscorea* spp.	*PDS*	Albino phenotype		Syombua et al., 2021
*Zea mays*	*LG1*	Smaller leaf angles		Li et al., 2017
*Lycium ruthenicum*	*FW2.2*	Variation in fruit size		Wang et al., 2021
*Humulus lupulus*	*PDS*	Albino phenotype		Awasthi et al., 2021
*Ocimum basilicum*	*DMR1*	Resistance to downy mildew		Navet and Tian, 2020
*Citrullus lanatus*	*PDS*	Albino phenotype		Tian et al., 2017
*Actinidia* Lindl.	*PDS*	Albino phenotype		Wang et al., 2018
*Solanum tuberosum*	*GBSS*	Increased amylopection/amylose ratio	Plasmid transfection of protoplasts	Andersson et al., 2017
*Malus prunifolia*	*PDS*	Albino phenotype	*Agrobacterium*-mediated transformation and protoplast transfection with RNPs	Osakabe et al., 2018
*Vitis vinífera*	*MLO-7*	Resistance to powdery mildew	Protoplast transfection with RNPs	Malnoy et al., 2016
*Citrus sinensis*	*PDS*	Albino phenotype	Xcc-facilitated agroinfiltration	Wang et al., 2014
*Citrus paradisi*	*LOB1*	Resistance to citrus canker	–	Jia et al., 2022
*Brassica oleracea*	*MYB28*	Increased glucoraphanin content	Protoplast transfection with RNPs	Kim et al., 2022

Similarly to most biotechnological developments, the first reports on using CRISPR in plants were “proof-of-principle” studies, *i.e.*, knocking out genes whose null phenotypes were easily observed. Most of them rely on phytoene desaturase (*PDS*) gene knockout, which disrupts the synthesis of carotenoids, resulting in an albino phenotype (Shan et al., 2013). Following these first publications on gene editing protocols for crop species, reports analyzing the phenotypical effects of specific genes began to be released. During the first decade (2013-2022), the technology was applied for various agronomical purposes.

For example, the knockout of *Oryza sativa* cytokinin oxidase/dehydrogenase (*OsCKX11*), an enzyme involved in cytokinin inactivation, was shown to result in a significant increase in branch, tiller and grain number compared with the wild type (Zhang et al., 2021). Transgenic barley, cassava, banana, soybean, rice, potato, and grapevine have been engineered to directly target DNA and RNA viruses. In contrast, cucumber and wheat plants have undergone editing of endogenous genes (host factors) for virus resistance (Robertson et al., 2022). In soybean, knockout of the *E1* gene resulted in the production of plants with early flowering under long-day conditions (Han et al., 2019). In banana, modifications in the *MaACO1* gene delayed natural fruit ripening from 21 days to 80 days, thus extending its shelf life (Hu et al., 2021). In sorghum, changes in an alpha-kafirin gene family increased digestibility and protein quality (Li et al., 2018). Low-gluten, non-transgenic wheat was developed via CRISPR engineering of the α-gliadin gene family (Sánchez-León et al., 2018), while a reduction in the toxic steroidal glycoalkaloids content in potato was achieved by knocking out the sterol side chain reductase 2 gene (Zheng et al., 2021).

Tomato has also been one of the most frequently edited species via CRISPR technology. For example, mutations in the *MAX-1* gene yielded plants resistant to the root parasitic weed *Phelipanche aegyptiaca* (Bari et al., 2021), while simultaneous knockout of the *SlINVINH1* and *SlVPE5* genes increased fructose and glucose levels for sweetness enhancement (Wang et al., 2021). Furthermore, mutating the *ENO* gene resulted in plants that yielded larger multilocular fruits (Yuste-Lisbona et al., 2020); knock-in of the salt-tolerant *SlHKT1;2* allele conferred tolerance to germination in 100 mM NaCl (Yuste-Lisbona et al., 2020); and single or multiple mutants for *SGR1*, *LCY-E*, *Blc*, and *LCY-B2* genes had their lycopene content increased (Li et al., 2018). Finally, mutations in the *SlAMS* gene promoted male sterility, which reduces the cost of F1 seed production (Bao et al., 2022).

Crop species are just beginning to be engineered via CRISPR. The next decade promises the generation of numerous agronomically interesting phenotypes via precise gene editing in many other species. Nevertheless, it is worth remarking that, despite the groundbreaking application of CRISPR in molecular plant breeding, only a few CRISPR-based crops have been approved for commercialization, such as soybean (Waltz, 2018), canola (Waltz, 2018), maize (Waltz, 2016), tomato (Waltz, 2022), and camelina (Waltz, 2018). Therefore, while scientists wield the needed molecular tool to sow the crop field, other social and legal hurdles remain to be overcome.

## 3 Plant life cycle in the context of genome editing

**Figure 2:**
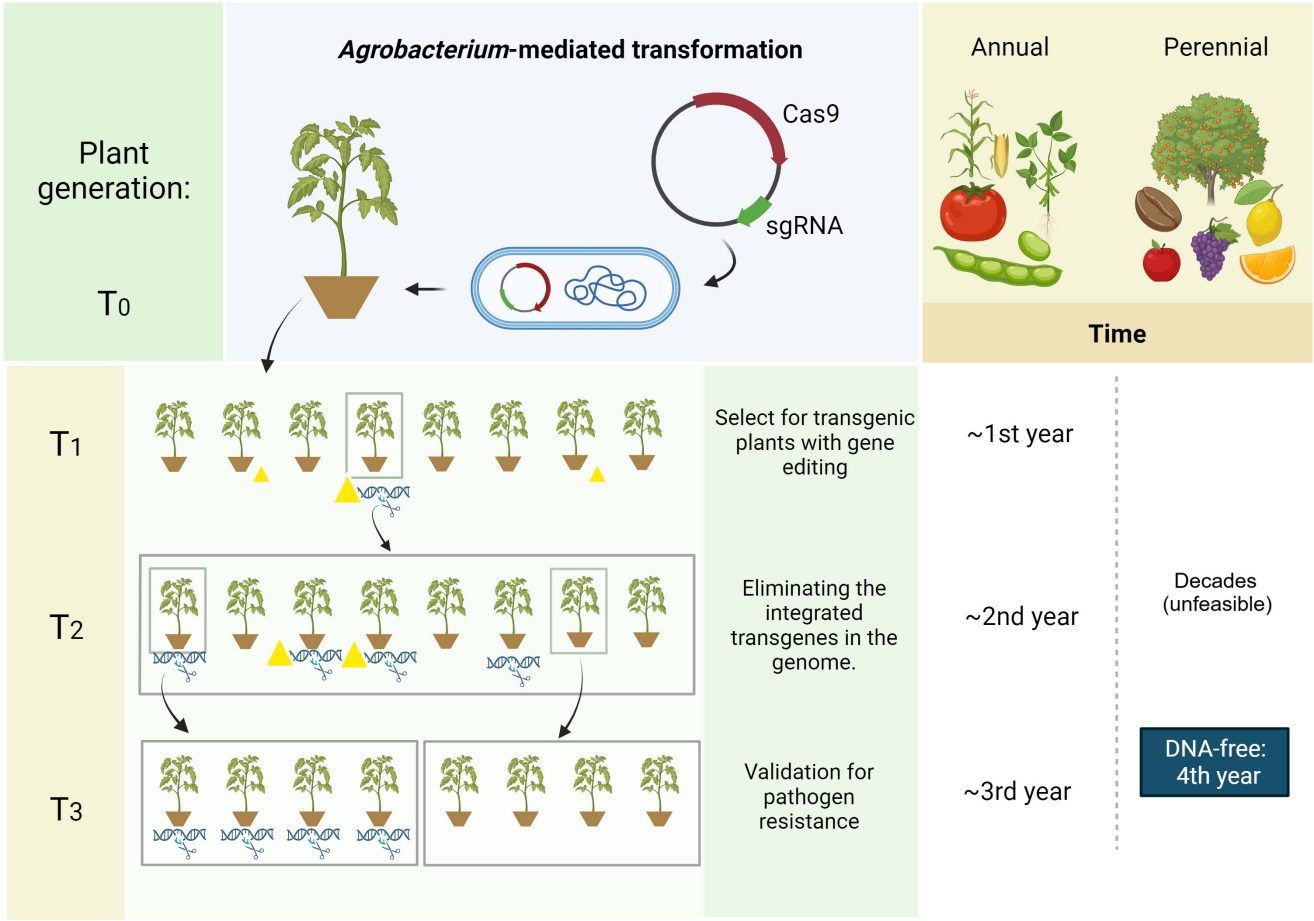
Plasmid-based CRISPR strategy for annual and perennial crops. Comparison of time needed for developing transgene-free events from annual and perennial crops when using a plasmid-based CRISPR strategy. Genetic transformation achieved using a transfection technique (e.g., *Agrobacterium tumefaciens*) yields CRISPR-edited transgenic plants in the T0 generation. Mendelian inheritance allows segregation for transgenes (yellow triangles) elimination while selecting for CRISPR-triggered mutation (DNA with scissor/cut DNA) over the next generations (T1, T2, T3). Plants can harbor transgenes without (triangle only) or with (triangle and cut DNA) gene editing, harbor only the gene editing without transgenes (cut DNA) or neither. Whereas annual crops can be selected for a DNA-free event within a few years, perennials usually take decades. In this case, DNA-free strategies (i.e., RNPs, IVTs, viral replicons) should be employed to accelerate this development process, possibly reducing the time to approximately four years, depending on the species. Image created with BioRender.com.

Crop plants can be categorized according to their life cycle lengths, such as annuals (e.g., soybean, maize), perennials (e.g., coffee, citrus, vines and the semi-perennial sugarcane), and biennials (e.g., beets, carrots, onions). Mostly, we see examples of CRISPR application in annual plants, such as cereals (Matres et al., 2021) and horticultural crops, such as tomato (Kumari et al., 2022). Among the characteristics that facilitate genome editing in annual plants is the rapid development of a new generation with the possibility of segregating the transgenes encoding the CRISPR/Cas system and other genetic elements from the gene construct in a short time (Lobato-Gómez et al., 2021) (**Figure 2**). Perennial plants, however, have a significant advantage, which consists of the possibility of fixing a mutation/editing that would hardly be carried forward in one generation (Lobato-Gómez et al., 2021). Also, due to the search for more sustainable agriculture, with greater potential for carbon fixation in the soil, there is an interest in converting traditionally annual crops into perennial ones, such as wheat (DeHaan et al., 2020). In these cases, genome editing has accelerated redomestication research, allowing the desired traits to be fixed in cultivars with better performance (Hanak et al., 2022).

Nonetheless, the long juvenile stage of perennial plants poses an additional obstacle when transgene segregation is needed due to delayed generational advancement (**Figure 2**), which usually takes up to decades for transgenes elimination aiming to keep CRISPR-induced mutations without the presence of plasmid backbone. Therefore, when considering this need, it is interesting to use a DNA-free method for delivering the editing machinery (Molinari et al., 2021). In this case, in order to ensure that the ribonucleoprotein complexes (RNPs) edit all genetic material (and not produce chimeras or mosaics), it is recommended that the particles are delivered into protoplasts (Woo et al., 2015). The difficulty of this procedure lies in the recalcitrance of protoplast regeneration in many plants (Reed and Bargmann, 2021). In these cases, other transgene-free methods can be used (Molinari et al., 2021), such as particle bombardment of either the mRNA-based CRISPR machinery (*in vitro* transcripts, IVTs) (Zhang et al., 2016) or ribonucleoproteins (Woo et al., 2015), as well as the use of virus-induced genome editing (VIGE) through viral replicon systems (Oh et al., 2021; Gentzel et al., 2022; Zhang et al., 2022) or transient expression via *Agrobacterium* spp. (i.e. agroinfiltration) (Kaur et al., 2021).

However, there are two major challenges when editing either annual or perennial plants. The first is its high heterozygosity rate, which makes it difficult to edit all gene alleles (Savadi et al., 2021). Prior sequencing of the target regions is necessary to circumvent this difficulty with the objective of a rational design of sgRNAs. Furthermore, since the regeneration process of post-transformation plants via *in vitro* tissue culture is highly dependent on the genotype, regeneration protocols still need to be established or even find recalcitrance in some plants (Chapman et al., 2022). In some cases, regeneration has been achieved by methods involving meristematic induction via genomic editing (Maher et al., 2020; Lian et al., 2022).

Regardless, the next topics provide an overview of the main parameters, achievements and bottlenecks for gene editing of three of the most economically important crops worldwide among perennial and semi-perennial plants. In this way, we could correlate and clearly show how plasmid-based strategies are being applied to them and how DNA-free approaches are promising and might overcome issues concerning transgene segregation and market feasibility.

## 4 Gene editing of citrus

The *Citrus* genus and related genera belong to the Rutaceae family and Aurantioideae subfamily (Talon et al., 2020), whose center of diversity extends from Tropical Africa through Southeast Asia and Eastern Australasia to Polynesia (Swingle and Reece, 1967). Sweet orange (*C. sinensis*) constitutes the most economically important citrus species in the world. In 2021, Brazil dominated global orange production, yielding approximately 16.2 million tons, followed by India and China (FAO, 2023). In addition to orange juice production, *Citrus* spp. are noteworthy for their potential in essential oil production (González-Mas et al., 2019) and pharmacological biomolecules (Ademosun et al., 2018).

**Figure 3:**
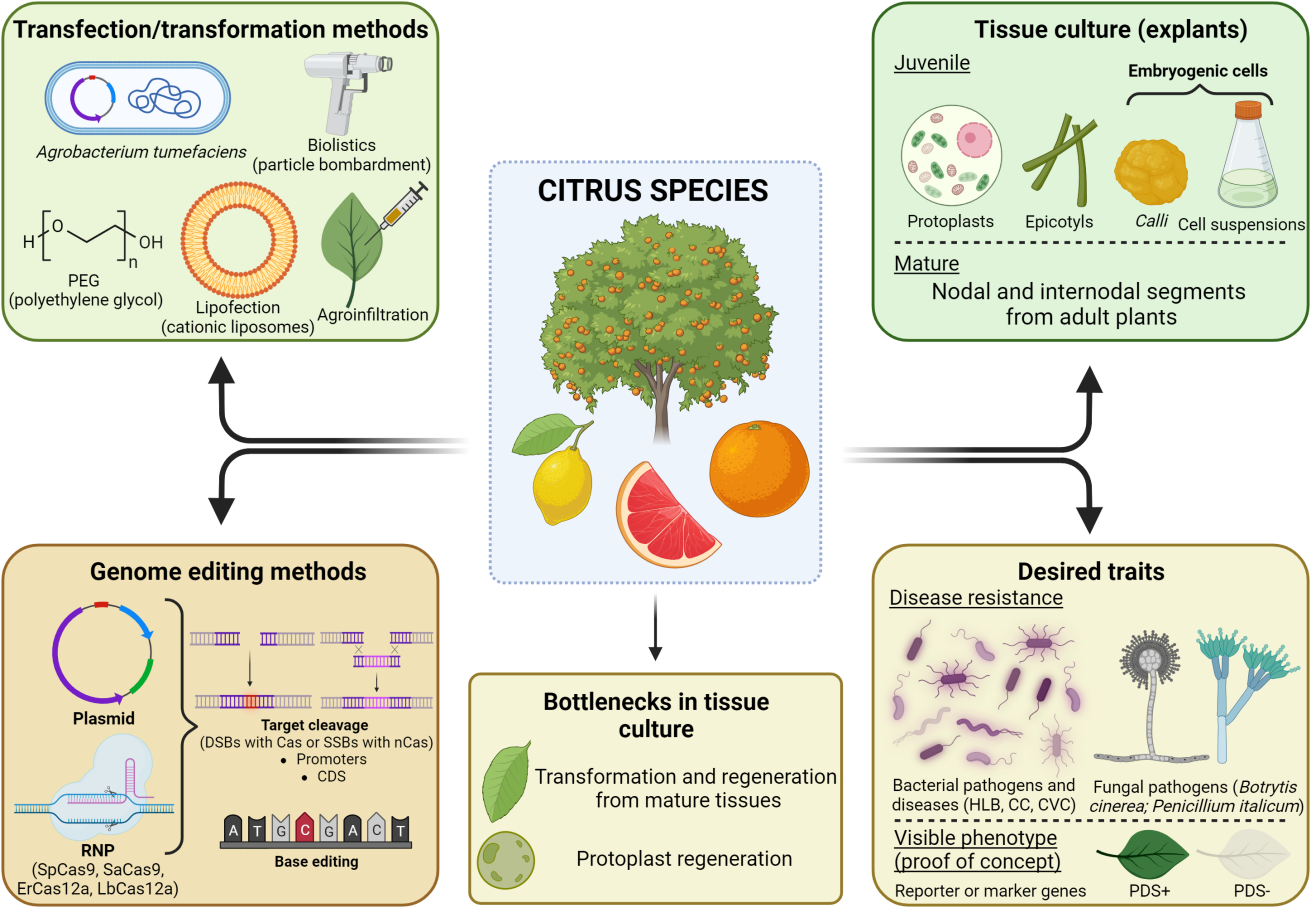
Citrus genome engineering scenario. Illustration of the genetic transformation methods already reported either for stable or transient expression, main explants (juvenile or mature) used for *in vitro* tissue culture, methods and strategies used for genome editing of citrus species, and the desired traits achieved through gene editing. Image created with BioRender.com.

Citrus species can be transformed through different techniques (Conti et al., 2021) (**Figure 3**), although *Agrobacterium*-mediated gene transfer is the most commonly used transformation method (Boscariol et al., 2006; Dutt and Grosser, 2009; Caserta et al., 2014; Souza-Neto et al., 2022). Different explants can be used to transform citrus species, mostly obtained from juvenile tissues, such as epicotyls and embryogenic cells, besides protoplasts (Omar et al., 2007; Dutt et al., 2020; Li et al., 2022; Mahmoud et al., 2022; Su et al., 2023). However, mature tissues are also possible sources of explants for citrus genetic engineering (Almeida et al., 2003; Peng et al., 2019). Li et al. (2022) have described the transformation of epicotyls in Carrizo citrange, whose explants were pre-treated with cytokinin (6-benzylaminopurine) and auxins (2,4-dichlorophenoxyacetic acid and 1-naphthaleneacetic acid), thus increasing the transformation efficiency from 11.5% to 31.8%.

Biolistics (particle bombardment) is also an alternative method, although less commonly used due to its low transformation efficiency (Yao et al., 1996; Wu et al., 2016; Wu et al., 2019). In order to increase its transformation efficiency and reduce shoot escapes, Boscariol et al. (2003) and Wu et al. (2019) developed and applied, respectively, a methodology based on *E. coli man*A as a selection marker gene. The *man*A gene confers to the transformed shoots the ability to metabolize mannose and its intermediates through the phosphomannose isomerase (PMI) enzyme (Stoykova and Stoeva-Popova, 2011).

The polyethylene glycol (PEG) method has been used for many years for citrus genetic transformation (Huang et al., 2020; Huang et al., 2022a; Su et al., 2023). Although PEG-based transformation has limitations related to cytotoxicity and low transformation efficiency (Vardi et al., 1990; Mahmoud et al., 2022), this method allows the transfection of exogenous macromolecules into the cell via endocytosis (Vardi et al., 1990; Couchoud et al., 2019). Based on this, sweet orange protoplasts were also edited through plasmid transfection mediated by cationic lipids (Mahmoud et al., 2022). The vector harboring sgRNA targeting the Nonexpressor of Pathogenesis-Related 3 gene (*CsNPR3*) was designed to promote a greater induction of systemic acquired resistance (SAR). For this, Mahmoud et al. (2022) used the technique called ‘lipofection’, which uses liposomes for the delivery of exogenous material. Liposomes fuse to the plasma membrane and their cargo is released into the cytoplasm. Lipofectamine LTX coupled to PLUS reagent composed the nanostructured vehicle that resulted in the highest transformation efficiency, keeping 90% of protoplasts viability. Furthermore, they verified that under the presence of the Arg9 CPPs (Cell-Penetrating Peptides), the transformation efficiency could be even more increased. Therefore, boosted cationic lipids may be an alternative to the use of PEG solution as a transfection agent for protoplasts and should be tested in plants for the transfection of RNP complexes. Electroporation is also a suitable alternative, since it does not have host range limitations. Nevertheless, in recent years, this technique has not been used for citrus transformation (Hidaka and Omura, 1993; Niedz et al., 2003).

In some techniques, transformation is not necessary for citrus genome editing (Alquézar et al., 2022; Huang et al., 2022b). Accordingly, Su et al. (2023) performed gene editing of the *CsLOB1* gene by using a DNA-free strategy based on RNPs, thus achieving a high rate of biallelic mutations and no off-target effect. In the same way, Huang et al. (2022) employed PEG-mediated transfection to trigger mutagenesis in protoplasts of *C. sinensis*, targeting the same gene. Additionally, they employed a highly efficient editing mechanism that modifies one or more base pairs through Cas nickase (nCas) with high specificity and low error rate (Azameti and Dauda, 2021; Molla et al., 2021).

It is known that citrus cultivars have a low regeneration rate and transformation efficiency (Peng et al., 2019; Wu et al., 2019). Furthermore, mosaicism during regeneration of citrus plants is a frequently reported issue (Alquézar et al., 2022), especially when epicotyls are used as explants. Due to these problems, protocols are often optimized for each variety (Dutt and Grosser, 2009; Oliveira et al., 2009) and, in order to minimize mosaicism rate, the use of embryogenic cells has been a feasible alternative to obtain engineered plants with a relatively high transformation efficiency (Dutt and Grosser, 2010; Dutt et al., 2020). Juvenility is another commonly faced problem for the evaluation of citrus phenotypes. Hence, in order to reduce flowering and fruiting time, Cervera et al. (2009) developed APETALA1 (AP1) transgenic citrange plants with short tree cycle which showed early flowering and fruiting. Low levels of polyembryony can also be a barrier to genetic transformation, since some species may produce a small number of nucellar embryos (Omar et al., 2007; Alquézar et al., 2017).

Regardless of the bottlenecks for genetic engineering and genome editing of citrus species, there are key traits that are interesting for genetic breeding that could already be improved or modulated in order to yield more desirable varieties. Disease resistance is one of the most important characteristics sought for citrus species. Huanglongbing/HLB (caused by *Candidatus* Liberibacter spp. – *C*Ls) and citrus canker (caused by *Xanthomonas citri* subsp. *citri* – *Xac*) are the main diseases in citrus orchards worldwide (Coletta-Filho et al., 2004; Ference et al., 2018; Bassanezi et al., 2020; Naqvi et al., 2022). Boscariol-Camargo et al. (2016) and Robertson et al. (2018) observed that transgenic plants overexpressing the Arabidopsis NPR1 protein displayed tolerance to HLB and resistance to citrus canker. Souza-Neto et al. (2022) observed that sweet orange and Carrizo citrange overexpressing the *mqsR* gene from *Xyllela fastidiosa* showed resistance to citrus canker and citrus variegated chlorosis. Potential genes of interest for genetic transformation and/or gene editing have been studied in order to obtain plants resistant to HLB (Curtolo et al., 2020), such as *AtPs21* and *CsACD2*, which act in repellency against the psyllid *Diaphorina citri* and as a susceptibility gene, respectively (Alquézar et al., 2017; Pang et al., 2020). Viral and fungal pathogens are important targets in citrus genetic engineering as well. Muccilli et al. (2020), for instance, developed a transgenic lemon expressing *chit12* gene, which confers tolerance to fungal pathogens in post-harvest conditions.

Abiotic stresses, such as drought and salinity, are also important problems faced in citrus orchards. In this case, genes coding for osmoprotectants (Longhi et al., 2022; Barichello et al., 2017) or transcriptional factors (Romero-Romero et al., 2020) can be interesting for genetic transformation or gene editing. In the field, modulation of genes involved in the architecture of the canopy and fruit quality (Dutt et al., 2022) are also interesting strategies to facilitate management and commercialization of citrus fruits.

Regarding achievements already made in citrus genome editing, reporter genes allowing easy identification of the resulting knockout phenotype, such as *CsPDS* (*C. sinensis* phytoene desaturase gene) (Jia and Wang, 2014; Jia et al., 2017a; Zhang et al., 2017; Dutt et al., 2020; Tang et al., 2021) and *CsALS* (*C. sinensis* acetolactate synthase gene) (Alquézar et al., 2022; Huang et al., 2022b), have been used to optimize gene editing protocols. Until now, the citrus species targeted for genome editing are Carrizo citrange (*Poncirus trifoliata* x *C. sinensis*), grapefruit (*Citrus paradisi*), pummelo (*Citrus maxima*) and sweet orange (*C. sinensis*). After optimizing these protocols, researchers have been editing target genes to mainly provide resistance against citrus canker. In this context, the *CsLOB1* (Citrus Lateral Organ Boundaries 1) gene has been the most studied because it encodes a transcription factor related to citrus canker susceptibility (Jia et al., 2016; Jia et al., 2017b; Peng et al., 2017; Huang et al., 2020; Jia and Wang, 2020; Huang et al., 2022a; Huang et al., 2022b; Su et al., 2023).

Jia et al. (2017b) obtained six independent Duncan grapefruit events edited for the *CsLOB1* gene, from which two (DLOB9 and DLOB10) did not develop pustules typical of citrus canker symptoms, and their mutation rates were the highest among the events (89.36% and 88.79%, respectively). In pummelo, eight transgenic events were obtained, but only those with homozygous and biallelic mutations showed resistance to citrus canker. The same was observed for Hamlin sweet orange events that have biallelic mutations in the EBE (Effector Binding Element) (Huang et al., 2022a) and TATA box regions (Huang et al., 2022b) of the *CsLOB1* gene promoter.

It is worth to mention that, although HLB is the most devastating citrus disease worldwide, there are no reports yet regarding resistant GE or even GM varieties. However, Curtolo et al. (2020) have identified key genes that are promising to achieve this trait, among them endochitinases B (*ChiB*) genes, which showed to be upregulated in a resistant pool made up from hybrids of *Citrus sunki* and *P. trifoliata* (an HLB-tolerant citrus species), and encoding vacuolar enzymes displaying putative antimicrobial activity (Medeiros et al., 2018) that should be evaluated against *C*Ls. Furthermore, HLB tolerance and resistance is also able to be achieved by disrupting susceptibility or sensitivity genes. In the latter case, Granato et al. (2019) observed that two out of nine callose synthase (*CsCalS*) genes, named *CsCalS7* and *CsCalS12*, were highly upregulated in *C. sinensis* during HLB infection and could significantly contribute to callose deposition in the phloem during bacterial colonization. This mechanism is known to trigger symptoms of this disease by blocking translocation of sap nutrientes, and could be enhanced when *CsCalS7* and *CsCalS12* are overexpressed. Hence, genome silencing of these genes could lead to symptoms attenuation or even avoid their arising, and edited plants could be HLB-tolerant.

Moreover, other sensitivity genes involved in the same mechanism of phloem obstruction during host response to bacterial infection are described in the literature and could be silenced as well. Batailler et al. (2012) and Ernst et al. (2012) have described the function of *SEO* (Sieve Element Occlusion) genes in encoding phloem proteins (P-proteins) that could aggregate and promote wound sealing in the sieve elements of *Arabidopsis thaliana* and *Nicotiana tabacum*, respectively. Based on this, Curtolo et al. (2020) evaluated *SEO* orthologs in *C. sinensis* and discovered that *SEOc* and *SEOd* genes were largely upregulated in all HLB-susceptible plants studied, as well as in some of the tolerant hybrids, thus indicating their analog function of phloem wound sealing by crystalloid proteins and suitability for gene silencing. Similarly, Boava et al. (2017) have discussed the function of the PP2 (Phloem Protein 2) gene family during HLB infection and found that these conserved phloem lectins encoded by *pp2* genes consequently block the transport of photoassimilates from sap to plant organs, leading to symptoms and tissues death, which thus suggests *pp2* genes as strong candidates for gene editing.

Regarding explants used for this intent, they are the same as those used for transgenic plant production (i.e. epicotyls, embryogenic cell suspensions and protoplasts) (**Table 2**). However, using epicotyls has the disadvantage of regenerating mosaic shoots for editing as well, as demonstrated by Zhang et al. (2017), which edited *PDS* gene and obtained mosaic, albino and green shoots from explants. Furthermore, the use of epicotyls depends on the availability of viable seeds. In contrast, shoots regenerated from embryogenic cell suspensions or protoplasts are derived from single cells, which eliminates mosaicism or chimerism (Dutt et al., 2020; Mahmoud et al., 2022).

**Table 2:** Summary of reports on citrus genome editing and details concerning genetic engineering and CRISPR/Cas parameters.

Species	CRISPR system*	CRISPR mechanism	Explants	Transfection technique	Target genes	Desired trait	Transformation efficiency	Editing rate	Reference
Carrizo citrange	hSpCas9	Plasmid	Epicotyls	*Agrobacterium tumefaciens*	Phytoene desaturase (*PDS*)	Albino phenotype	no data	45.5% - 75%	Zhang et al., 2017
Carrizo citrange	SaCas9	Plasmid	Epicotyls	*Agrobacterium tumefaciens*	Cs7g03360 (*Ago7*)	Leaf development abnormality	no data	15.55% - 79.67%	Jia et al., 2017a
Carrizo citrange	pcoCas9	Plasmid	Epicotyls and protoplasts	PEG solution and *Agrobacterium tumefaciens*	Lateral organ boundaries 1 (*CsLOB1*)	Citrus canker resistance	no data	44.4%	Huang et al., 2020
Carrizo citrange	nSpCas9	Plasmid	Epicotyls	*Agrobacterium tumefaciens*	Acetolactate synthase (*CsALS*)	Resistance to herbicide IMZ	no data	11.7%	Alquézar et al., 2022
Carrizo citrange	npcoCas9	Plasmid	Epicotyls	*Agrobacterium tumefaciens*	Acetolactate synthase (*CsALS*)	Resistance to herbicide IMZ	no data	no data	Huang et al., 2022b
Ducan grapefruit	SpCas9	Plasmid	Epicotyls	*Agrobacterium tumefaciens*	Lateral organ boundaries 1 (*CsLOB1*)	Citrus canker resistance	no data	no data	Jia et al., 2016
Ducan grapefruit	SaCas9	Plasmid	Leaves	*Xcc-facilitaded agroinfiltration*	Phytoene desaturase (*PDS*) and Cs2g12470	Albino phenotype	no data	no data	Jia et al., 2017a
Ducan grapefruit	SpCas9	Plasmid	Epicotyls	*Agrobacterium tumefaciens*	Lateral organ boundaries 1 (*CsLOB1*)	Citrus canker resistance	no data	no data	Jia et al., 2017b
Ducan grapefruit	npcoCas9	Plasmid	Epicotyls	*Agrobacterium tumefaciens*	Lateral organ boundaries 1 (*CsLOB1*)	Citrus canker resistance	no data	no data	Huang et al., 2022b
Pummelo	pcoSpCas9	Plasmid	Epicotyls	*Agrobacterium tumefaciens*	Lateral organ boundaries 1 (*CsLOB1*)	Citrus canker resistance	no data	no data	Jia and Wang, 2020
Sweet orange	SpCas9	Plasmid	Leaves	*Xcc-facilitaded agroinfiltration*	Phytoene desaturase (*PDS*)	Albino phenotype	no data	no data	Jia and Wang, 2014
Sweet orange	pcoCas9	Plasmid	Epicotyls	*Agrobacterium tumefaciens*	Lateral organ boundaries 1 (*CsLOB1*)	Citrus canker resistance	no data	34.54%	Peng et al., 2017
Sweet orange	AtCas9	Plasmid	Embryogenic cell suspensions	*Agrobacterium tumefaciens*	Phytoene desaturase (*PDS*)	Albino phenotype	26.3% - 36.5%	no data	Dutt et al., 2020
Sweet orange	hSpCas9	Plasmid	Epicotyls	*Agrobacterium tumefaciens*	Phytoene desaturase (*PDS*)	Albino phenotype	18.22% - 21.15%	no data	Tang et al., 2021
Sweet orange	pcoCas9	Plasmid	Protoplasts	Cationic lipids	Nonexpression of pathogenesis-related 3 (*CsNPE3*)	Induction of acquired systemic resistance	no data	no data	Mahmoud et al., 2022
Sweet orange	pcoCas9	Plasmid	Epicotyls and protoplasts	PEG solution and *Agrobacterium tumefaciens*	Lateral organ boundaries 1 (*CsLOB1*)	Citrus canker resistance	no data	no data	Huang et al., 2022a
Sweet orange	npcoCas9	Plasmid	Epicotyls	*Agrobacterium tumefaciens*	Lateral organ boundaries 1 (*CsLOB1*)	Citrus canker resistance	no data	no data	Huang et al., 2022b
Sweet orange	ErCas12a and LbCas12aU	RNP	Protoplasts	PEG solution	Lateral organ boundaries 1 (*CsLOB1*) and *PDS*	Citrus canker resistance and albino phenotype	no data	no data	Su et al., 2023
Sweet orange	LbCas12a	RNP	Protoplasts	PEG solution	Plasma membrane ATPAse (*CsPH5*)	Plasma membrane ATPase editing	no data	35.3% - 90.8%	Zhang et al., 2022

*pcoCas9, plant codon-optimized SpCas9; nCas9, Cas9 nickase; npcoCas9, Cas9 nickase Cas9 from dicot codon-optimized Cas9.

In general, plasmid vectors used in citrus editing harbor kanamycin or hygromycin selectable marker genes, as well as employ *GUS* or *GFP* as reporter genes. Multiplex editing systems are mostly based on Csy4 endoribonuclease or polycistronic tRNA-gRNA for sgRNA units processing (Jia and Wang, 2014; Jia et al., 2017a; Zhang et al., 2017; Dutt et al., 2020; Huang et al., 2020; Tang et al., 2021; Alquézar et al., 2022; Huang et al., 2022a; Huang et al., 2022b). Yang et al. (2023) optimized the transfection of callus-derived protoplasts through the PEG method, evaluating multifactorial conditions, and proved the functionality of the polycistronic tRNA-gRNA system in protoplasts for transient expression. Huang et al., 2020 demonstrated that in Carrizo citrange the tRNA-gRNA multiplex system had a higher editing efficiency than the Csy4 system, possibly due to the fact that the tRNA-gRNA system depends on an endogenous processing machinery (i.e. native tRNA expressed by the host), in contrast to the heterologous processing machinery provided when using the Csy4 system. Nevertheless, Dutt et al. (2020) edited the *CsPDS* gene in embryogenic cell suspensions using two sgRNAs through the Csy4 processing system. The transformation rate obtained was 36.5% and from the 12 events evaluated, all of them were edited by sgRNA1 and 11 had mutations triggered by sgRNA2, thus demonstrating high editing efficiencies. As expected, they also demonstrated the absence of mosaic embryos due to the use of cell suspensions.

Concerning the CRISPR system chosen, plant codon-optimized SpCas9 (*Streptococcus pyogenes* Cas9) is a recommended nuclease to be used, but Jia et al., 2017a demonstrated that it is possible to perform gene editing in Carrizo citrange and Duncan grapefruit using the native Cas9 from *Staphylococcus aureus* (SaCas9). This nuclease has the advantage of reducing the number of off-targets due to its stringent PAM sequence (NNGRRT). In the genome of *C. sinensi*s, for example, the SaCas9 PAM occurs every 79 bp, whereas the SpCas9 PAM occurs every 32 bp, which makes SaCas9 a more specific nuclease for its gene editing.

Additionally, efforts have been made to develop protocols that allow DNA-free genome editing. This approach is very useful because it facilitates the release of genetically modified events by circumventing regulatory issues related to GM development (Ishii and Araki, 2016), since mutations occur without inserting exogenous DNA into the host genome (Molinari et al., 2021). For this, *C. sinensis* protoplasts have been transfected with three subtypes of Cas nucleases composing RNPs: ErCas12a, LbCas12a and LbCas12aU (Zhang et al., 2022; Su et al., 2023). Zhang et al. (2022) tested different RNP concentrations using LbCas12a nuclease to edit the *CsPH5* gene and concluded that 0.1 µM allows the best editing efficiency (90.8%). Su et al. (2023) performed a protocol for developing DNA-free edited plants within 10 months and showed that both ErCas12a and LbCas12aU were efficient to generate biallelic/homozygous *CsPDS* mutations.

## 5 Gene editing of coffee

Coffee is one of the most consumed beverages worldwide and its production reached a volume equivalent to 167 million bags of 60 kg in the year 2020-2021 (https://www.embrapa.br/). Coffee plants belong to the genus *Coffea*, being *Coffea arabica* and *Coffea canephora* the main species responsible for the production of grains consumed all over the world. *C. canephora* species is diploid, allogamous, highly productive and resistant to some pests and diseases. On the other hand, *C. arabica* is an allopolyploid (4n) resulting from a natural hybridization between *C. canephora* and *C. eugenioides*, preferentially autogamous, susceptible to several pathogens, and highly productive. Arabica grains result in a high cup quality, including specialty coffees (Carvalho et al., 1991).

Breeding of *Coffea* species guaranteed the availability of commercial cultivars adapted to different environments, easily managed, highly productive, resistant to biotic and abiotic stress, and most importantly with high organoleptic quality and specific chemical attributes. However, traditional coffee breeding is time-consuming and limited by the low genetic diversity of *C. arabica* germplasm. Therefore, the use of novel genome-based methodologies, such as MAS and genome editing, represents an opportunity to introduce novel traits into this culture in a faster and more controlled manner (Guerreiro-Filho and Maluf, 2019).

The *in vitro* cultivation of *Coffea* species started in the early 1970s, with the development of protocols of somatic embryogenesis, aiming to use *in vitro* strategies to multiply coffee seedlings from elite cultivars, special hybrids and potential F1 (Campos et al., 2017). Later, with the advancements in plant transformation, embryogenic callus could be selected for plant regeneration (Etienne et al., 2018). Those studies also indicated that embryogenesis and regeneration efficiency depend on the genetic background, media composition, type of explants and cultivation conditions.

Indirect somatic embryogenesis is the most promising tissue culture technique in coffee, since regeneration into viable adult plants is well-established, although it still needs to be optimized for large-scale production of seedlings (Mishra and Slater, 2012; Etienne et al., 2018). However, embryogenic calli are suitable for coffee transformation. Ribas et al. (2011) compared different cultivation and selection methods and determined that a four-month established embryogenic callus culture has a high transformation efficiency. Also, they found that color and type of callus, as well as culture media composition, are key parameters to improve transformation efficiency.

**Figure 4:**
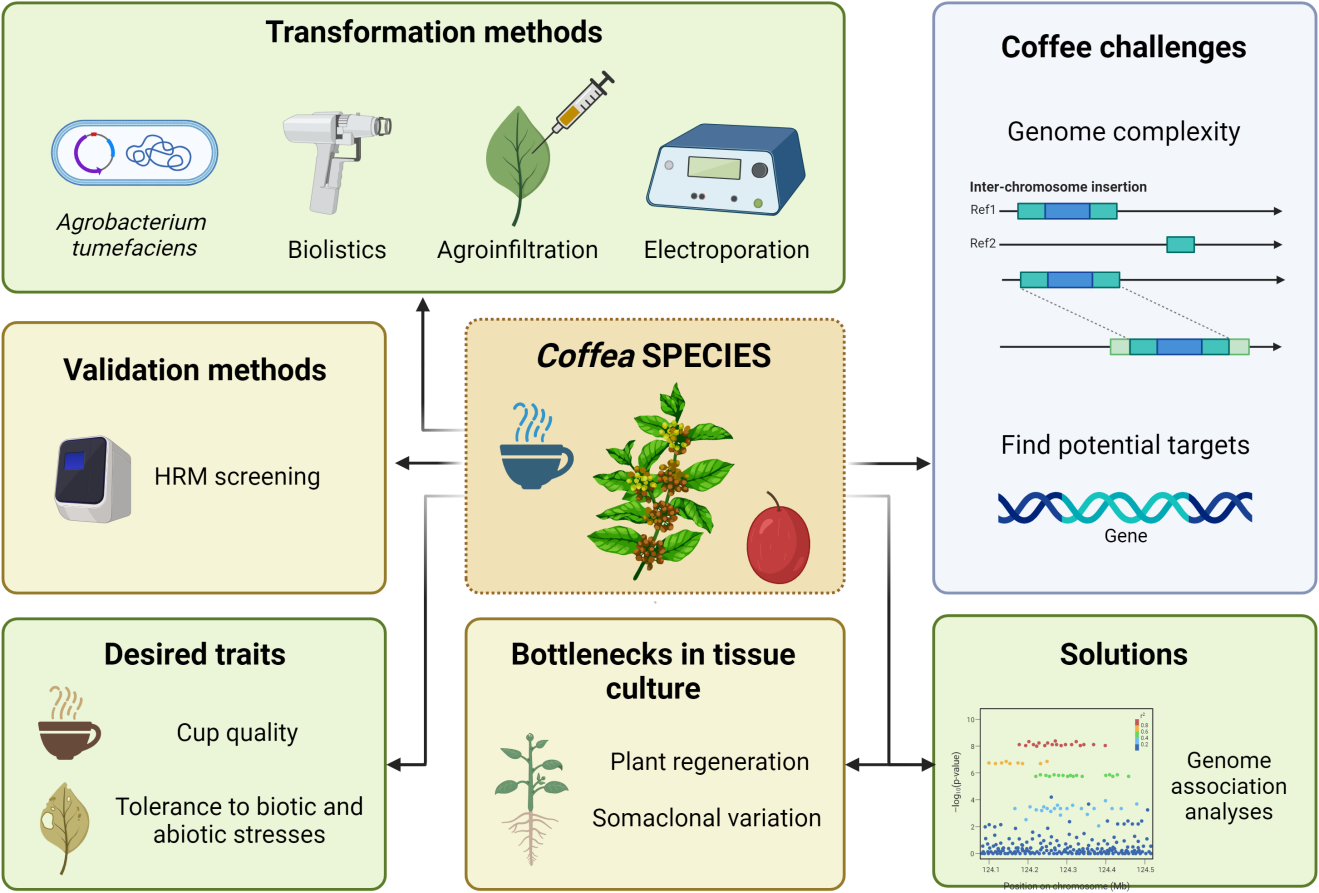
Coffee genome engineering scenario. Illustration of the genetic transformation methods reported, the genomic complexity and low number of identified gene targets as major challenges, the low regeneration rate as a limitation on *in vitro* tissue culture, the genome-wide association studies as a strategy to identify genes associated with agronomic traits, and the possibilities on coffee genome editing. Image created with BioRender.com.

Methods for efficient transformation of both *C. arabica* and *C. canephora* species mostly include particle bombardment (Ribas et al., 2005; Albuquerque et al., 2009; De Guglielmo-Cróquer et al., 2010), *Agrobacterium*-mediated transformation of embryogenic callus (Ribas et al., 2011), hairy roots (Alpizar et al., 2006), agroinfiltration of mature leaves (Vargas-Guevara et al., 2018) and protoplast electroporation (Fernandez and Menéndez-Yuffá, 2003) (**Figure 4**). Overall transformation efficiency depends on several parameters, reviewed by Mishra and Slater (2012) and Etienne et al. (2018), and is comparable to other plant species. Despite these initiatives, coffee transformation is not a regular strategy, neither for the development of novel cultivars nor to evaluate the effect of any given gene over selected traits. Major limitations for the use of transformation in coffee culture include the allopolyploid nature of *C. arabica*, the long-life cycle and high cost to maintain evaluation areas. Also, another major drawback is the long and laborious process of coffee *in vitro* regeneration, which does not always result in mature transformed plants. On the other hand, molecular analysis indicated that regenerated transformed plants display very low or no somaclonal variation (Landey et al., 2013; Oliveira et al., 2019).

Most of the genetic transformation events of *Coffea* obtained so far aimed to develop plants resistant to diseases and pests. To introduce the *Cry1Ac* gene of *Bacillus thuringiensis* (Bt) into *C. arabica*, biolistics was used in somatic embryos to confer resistance to *Leucoptera coffeella*. However, out of a total of 12 embryos, only one expressed the gene of interest (De Guglielmo-Cróquer et al., 2010). The same gene was also successfully introduced into *C. canephora* through *A. tumefaciens* (Perthuis et al., 2005). Looking for resistance to the coffee berry borer (CBB), *Hypotheneumus hampei*, Valencia-Lozano et al. (2021) developed transgenic *C. arabica* var. Typica expressing Bt Cry10Aa by biolistics. The authors achieved 16.7% transformation efficiency, and seeds harvested from the T1 generation, derived from three transformed plants, expressed Cry10Aa and successfully controlled CBB. Fruits of genetically transformed plants inhibited the development and infestation capacity of CBB females (Valencia-Lozano et al., 2021). Similarly, particle bombardment was used to transform embryogenic calli of *C. arabica* with the α-amylase-1 inhibitor gene (*α-AI1*) that triggers resistance to CBB (Albuquerque et al., 2015). The authors evaluated 54 plants and observed that the *α-AI1* transgene was stably inherited in the T2 progeny and expressed in a tissue-specific manner in seeds by using the PHA-L promoter. Despite these efforts, there is no GM coffee cultivar available for cultivation yet.

Regardless of the limitations of *in vitro* tissue culture and the genetic transformation described here, genome editing of coffee represents a promising strategy to develop novel cultivars. Breeding perennial plants is time-consuming, and the possibility to modify specific traits by editing target genes with no other potential change in the genetic background is very appealing. In coffee, gene editing studies available so far (**Table 3**) are merely proof of concepts in *C. canephora* plants (Breitler et al., 2018; Casarin et al., 2022). In both studies, *PDS* was used as a target gene and the CRISPR machinery was delivered to embryogenic callus by *A. tumefaciens*. The overall editing efficiencies were 30.4% (Breitler et al., 2018) and 76.9% (Casarin et al., 2022), with 7.6% and 54% of homozygous mutations, respectively. Besides albine and variegated seedlings, Casarin et al. (2022) also observed seedlings with abnormal cotyledon and root development, and limited growth. Based on the mutation rates obtained in those studies, the use of editing strategies by breeding programs is encouraging. However, editing of *C. arabica* remains a challenge due to its allopolyploid nature, which means that four alleles must be edited simultaneously to obtain a homozygous mutated trait.

**Table 3:** Summary of reports on coffee genome editing and details concerning genetic engineering and CRISPR/Cas parameters.

Species	CRISPR system	CRISPR mechanism	Explants	Transfection technique	Target gene	Desired trait	Transformation efficiency	Editing rate	Reference
*Coffea canephora*	NLS-Cas9	Plasmid	Embriogenic callus	*Agrobacterium tumefaciens*	Phytoene desaturase (*PDS*)	Albino phenotype	no data	30.4%	Breitler et al., 2018
*Coffea canephora*	NLS-Cas9	Plasmid	Embriogenic callus	*Agrobacterium tumefaciens*	Phytoene desaturase (*PDS*)	Albino phenotype	no data	76.9%	Casarin et al., 2022

Nonetheless, CRISPR technology remains an important and strongly promising tool to improve the quality of coffee beverage by reducing caffeine content, among other desired traits. In this way, genomic selection and association studies have been useful to accelerate molecular breeding and to reduce the delivery time of more adapted coffee cultivars in *C. arabica* (Carvalho et al., 2020; Sousa et al., 2021; Carvalho et al., 2023) and *C. canephora* (Ferrão et al., 2018; Adunola, et al., 2023). Advances in scale, resolution and analysis of “omics” techniques help to reveal possible targets for genome editing. However, high-impact coffee challenges such as drought and heat tolerance, as well as resistance against CBB and to aggressive nematodes such as *Meloidogyne* spp. and *Pratylenchus* spp. require further study of the molecular mechanisms behind their regulation and plant interactions. Anyway, we can point out interesting traits for coffee breeding that may be a closer reality due to the availability of potential targets for genome editing.

Concerning biotic stresses (i.e., pest control and disease resistance), the coffee leaf miner (CLM), *L. coffeela*, is a major concern for *Coffea* spp. It is a monophagous insect whose caterpillar feeds on the coffee leaves causing great losses by reducing its productivity. Resistance to CLM derived from *Coffea racemosa*, a moderately resistant species used in Brazilian breeding programs, is controlled by two complementary dominant genes (Guerreiro-Filho et al., 1999). Microarray analysis was used to unravel the molecular basis of plant mechanisms involved in coffee responses to CLM attacks (Cardoso et al., 2014). The study provides information on molecular aspects of CLM defense mechanisms, describing pathways regularly activated in response to herbivory, primary and secondary metabolism pathways and the expression of genes related to plant antibiosis strategy. The authors concluded that differential expression profiles between resistant and susceptible genotypes are observed in the absence of the leaf miner, indicating that the defense is already built into resistant plants as a priming mechanism. In addition, some potential marker candidate genes were validated by RT-qPCR (Cardoso et al., 2014).

Moreover, GWAS was carried out on an arabica coffee population derived from *C. racemosa* used for studies on CLM resistance (Nonato et al., 2021). The authors identified four SNPs significantly associated with jasmonic acid metabolism and with LRR-RLK proteins. The first one has an important role in resistance to biotic agents, and the second recognizes pathogen-associated molecular patterns (PAMPs) and herbivore-associated molecular patterns (HAMPs), thus suggesting potential mutagenesis target sites to be mimicked towards achieving CLM resistance in coffee.

Coffee leaf rust (CLR), caused by *Hemileia vastatrix*, and coffee berry disease (CBD), caused by *Colletotrichum kahawae*, are diseases limiting coffee productivity. CLR is present in coffee producing regions around the world (Ventura et al., 2019), whereas CBD is restricted to *C. arabica* in Africa (Van der Vossen and Walyaro, 2009). According to Ventura et al. (2019), genetic resistance to *H. vastatrix* is observed in diploid species such as *C. canephora*, *Coffea congensis*, *Coffea dewevrei* and *Coffea liberica*, and is conditioned by at least one of nine dominant genes (*S_H_1* to *S_H_9*). Knowledge about the molecular basis of the mechanisms involved in resistance to *H. vastatrix* advanced through proteomics by identifying markers of resistance to this fungus (Guerra-Guimarães et al., 2015) and transcriptome analyses of the plant-pathogen interaction (Castro et al., 2022; Estanislau, 2022).

Targeting to speed up the breeding programs, DNA markers for CRL and CBD resistance have already been suggested for marker-assisted selection of *C. arabica* genotypes with introgressed genes from other coffee species (Silva et al., 2018) and for characterization of germplasm diversity (Alkimim et al., 2017). Nonato et al. (2021) found five candidate genes close to SNPs significantly associated with leaf rust according to the type of reaction and type of fungal lesion. Three genes (*T*, *R* and *k*) are involved in resistance to CLB (Van der Vossen and Walyaro, 1980). In addition, Gimase et al. (2020) used GWAS and found associations between two SNP markers (Ck-2 and CK-3) with CBD resistance.

Concerning marketing and quality traits, sensory attributes of coffee beans are influenced by the chemical composition of green beans, which in turn is controlled by genetic factors (Ky et al., 2001; Leroy et al., 2006; Farah, 2009; Tran et al., 2017), environment production, fruit maturation physiology and technological factors such as post-harvest processing (Kitzberger et al., 2020; Wondimkun et al., 2022) or even by different methods of coffee brewing (Novaes et al., 2019; Chavez et al., 2022). It is known that within the species *C. arabica* (Ky et al., 2001; Scholz et al., 2016) and *C. canephora* (Mori et al., 2016) there is genetic variability for non-volatile biochemical precursors of coffee aroma such as caffeine and trigonelline, chlorogenic acids, sucrose, and lipids such as cafestol and kahweol diterpenes (Ky et al., 2001; Farah, 2009). GWAS studies performed by Sant’Ana et al. (2018) identified five SNPs associated to lipid content: four with cafestol, three with kahweol and nine with the cafestol/kahweol ratio. As most of these SNPs are located inside or near genes from the metabolic pathways of these chemical compounds in coffee beans, they are potential targets for gene editing approaches.

Among other potential targets for editing in coffee, genes promoting caffeine synthesis are the most promising candidates. A naturally decaffeinated beverage is a recurrent demand from a growing market that seeks coffees with special organoleptic characteristics. Although caffeine is known for its stimulant effects, it can also cause, in sensitive people, unwanted responses such as headaches, tremors and nausea, among others. Caffeine synthesis in coffee is controlled by three genes encoding methyltransferases responsible for converting xanthine into caffeine (Ashihara et al., 2008), which have a simple genetic inheritance (Favoretto et al., 2017).

Caffeine accumulates in all coffee tissues, and since early developmental plant stages, what makes it a valuable biochemical marker. Molecular analysis of a naturally caffeine-free *C. arabica* plant indicates that the lack of caffeine does not affect other agronomic traits (Guimarães et al., 2021). In this study comparing large-scale gene expression from branches, buds and fruits from regular and caffeine-free coffee plants, the authors identified 171 transcripts out of 65,000 presenting differential expression between both groups. *In silico* analysis indicated that most of these transcripts are unrelated to caffeine metabolism or plant physiology impairment. Therefore, blocking caffeine synthesis in coffee fruits through gene editing represents a promising strategy to develop caffeine-free events.

Previous studies attempted to block caffeine synthesis through downregulation of the first gene of the caffeine biosynthetic pathway, coding for xanthosine methyltransferase (*MXMT*), by using RNAi. This strategy led to a 50-70% reduction in caffeine content in transgenic *C. canephora* leaves, and an almost abolishment of caffeine content in embryogenic tissues of *C. arabica* (Ogita et al., 2003). As a result of this strategy, a 20% increase in theobromine content and a reduction in transcripts from the *XMT* (theobromine synthase) and *DXMT* (caffeine synthase) genes were also observed. Later, the same genetic engineering approach was used to decrease caffeine content by targeting the N-methyl transferase gene family, but this effect was shown to be ineffective over time (Mohanan et al., 2014). Despite the promising results at the seedling stage, none of those events resulted in a mature caffeine-free coffee plant. However, similar approaches based on CRISPR/Cas technology can also be used to achieve this aim in an effective way, thus promoting gene silencing at the genomic level.

## 6 Gene editing of sugarcane

Sugarcane (*Saccharum* spp.) is a C4 grass crop that probably originated in Southeast Asia and New Guinea, where its domestication occurred about 10,000 years ago (Lebot, 1999). Its global economic importance is bolstered by its use as a main feedstock in the production of sugar, bioenergy, and other valuable by-products (e.g., bioplastics, forage). To illustrate, the global sugarcane production in 2020 was around 1.8 billion tons, headed by Brazil, India, and China (FAOSTAT, 2022). Therefore, its enormous potential as a bioenergy feedstock, which is supported by its high photosynthetic efficiency, underscores the importance of sugarcane breeding programs.

Sugarcane breeding programs generally focus on the genetic improvement of major production traits, such as cane yield, biomass and fiber outcomes, sucrose accumulation, uniform tillering, and better stem elongation (Gouy et al., 2013). However, as climate change intensifies, causing severe harm to sugarcane crops (Linnenluecke et al., 2018), other features, such as those providing drought tolerance, have been within the scope of breeders. These features include deep root systems, stay-green phenotype, and erect canopies (Meena et al., 2022), striving for climate-resilient, high-yielding crops. Nevertheless, agronomically essential traits are complex and require genome-wide DNA marker approaches for their genomic predictions (Hayes et al., 2021).

Among other issues threatening sugarcane production and reducing biomass and its by-products is the severity of some pests and diseases. Sugarcane grows well in tropical and subtropical regions in conditions that are also optimal for establishing a range of pathogens (Sanguino, 1998). The major fungal diseases influencing productivity to a greater or lesser extent in different regions are smut, brown and orange rusts, brown spots, pineapple rot, red rot, and fusariosis (Verma et al., 2022). The most common examples of bacteriosis are leaf scald and ratoon stunt disease (Monteiro-Vitorello et al., 2009; Barbasso et al., 2010; Sundar et al., 2015). Also noteworthy, pests such as Plant Parasitic Nematodes (PPN) that attack the roots of living plants have been responsible for widespread yield losses across all sugarcane fields of different soil compositions (Dinardo-Miranda et al., 2019). Besides their regular presence, climate change will impact population dynamics and the overall occurrence of pests and pathogens, contributing to losses in productivity and affecting sugarcane growth and metabolism (Velásquez et al., 2018).

Developing resistant genotypes is the most reliable and durable way to secure plants from pathogens. Beyond the search for ‘major resistance genes’ (*R* genes), for most diseases, quantitative resistance has many advantages (Pilet-Nayel et al., 2017). Diagnostic markers for quantitative resistance contemplate investigating variation in genes involved directly in pathogen recognition or related processes (‘candidate gene approach’) or an untargeted method such as comparing RNA-Seq data of resistant versus susceptible plants (Mosquera et al., 2016). Furthermore, the exploration of ‘susceptibility genes’ (*S* genes) with subsequent gene knockout can enhance plant resistance against specific pathogens by abolishing their compatibility with the host (Moniruzzaman et al., 2020). Therefore, the integrated use of modern approaches, such as next-generation sequencing (NGS) and genome editing, might contribute to the prospection of candidate genetic elements for developing high-performance gene-edited sugarcane crops.

The sugarcane genome is huge (>10 gigabases) and has the most polyploidy (2n = 100-120) known among domesticated species (Piperidis and D’Hont, 2020), even with events of aneuploidy, inter-chromosomal translocations, and hybridization between species. The homologous chromosomes of modern commercial hybrids originated mainly from *Saccharum officinarum* (2n = 80, x = 10) and *Saccharum spontaneum* (2n = 40–128, x = 8), whose genomes contributions correspond to 80% and 10-15%, respectively (D'Hont et al., 1996; Garsmeur et al., 2018; Piperidis and D’Hont, 2020). Undoubtedly, the complex sugarcane genome represents an obstacle to crop improvement through classical or biotechnological genetic breeding. Additionally, conventional breeding programs face challenges such as dependence on photoperiod and temperature condition in floral induction, lack of pollen fertility and flowering synchrony in specific crosses (Melloni et al., 2015), besides a narrow genetic background (Berding and Roach, 1987). Consequently, it is very costly, extremely laborious, and time-consuming, taking 10–15 years to release a new elite variety (Gazaffi et al., 2010). In contrast, *Saccharum* vegetative propagation is a feature that favors the improvement of cultivars by conventional transgenesis and genome editing approaches, which overcomes the sexual barriers, difficulties of outcrossing *Saccharum* species, and transmits intended modifications into the genome without segregation by sexual reproduction (Ingelbrecht et al., 1999; Oz et al., 2021).

**Figure 5:**
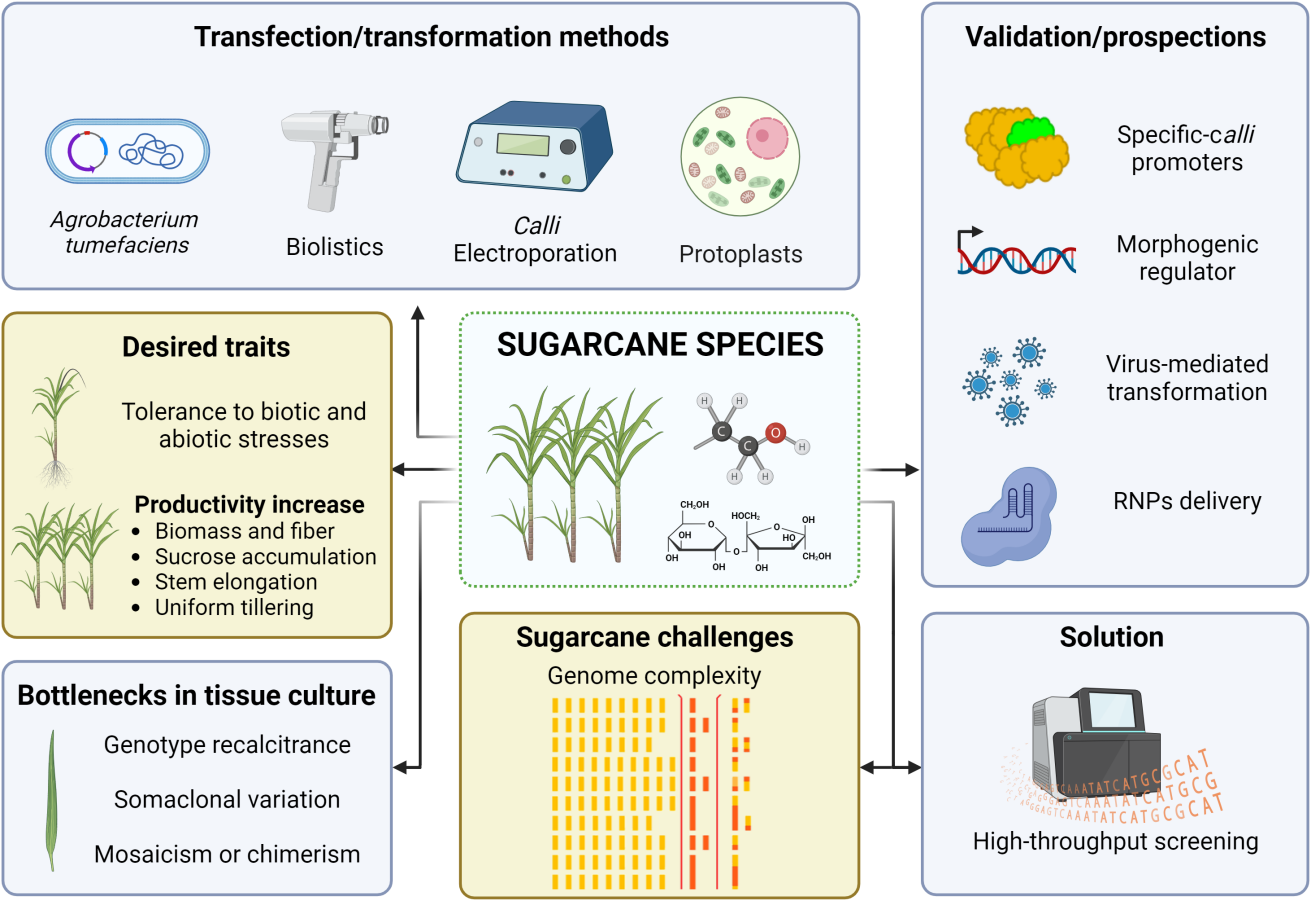
Sugarcane genome engineering scenario. Illustration of the genetic transformation methods reported, the genomic complexity as a major challenge, the issues on *in vitro* tissue culture, the high-throughput screening of gene-edited events as a solution, and the prospected strategies for sugarcane genome editing. The genome complexity icon was adapted from Garsmeur et al. (2018). Image created with BioRender.com.

While in the mid-1990s the world witnessed a set of approved transgenic crops (James and Krattiger, 1996), two decades passed until the first commercial approval of a transgenic sugarcane crop in 2017. Sugarcane genetic transformation protocols emerged in the 1990s, when the first transgenic sugarcane plant, carrying a selectable marker gene (*neomycin phosphotransferase - nptII*), was developed using particle bombardment of embryogenic callus (Bower et al., 1996). Sugarcane transformation mediated by intact cell (calli) electroporation was sparse and temporarily reported in the literature (Arencibia et al., 1992; Arencibia et al., 1995; Arencibia et al., 1997). On the other hand, the *Agrobacterium tumefaciens*-mediated transformation was successfully achieved by Arencibia et al. (1998), using the reporter gene *gus* (*uidA*) to optimize the protocol. Shortly thereafter, *Agrobacterium*-mediated transformation using the bacterial PPT acetyltransferase gene (*bar*) rendered glufosinate-resistant sugarcane plants (Enríquez-Obregón et al., 1998). Further on, highly efficient protocols have emerged and fostered the industrial-scale production of transgenic plants (Dong et al., 2014; Basso et al., 2017). Furthermore, there are limited reports on sugarcane protoplast transformation that, however, have left the regeneration step as an unsolved problem (Chen et al., 1987; Rathus and Birch, 1992). Thus, transformation via particle bombardment and *A. tumefaciens* of embryogenic callus or leaf rolls are the most widespread approaches (**Figure 5**) (Budeguer et al., 2021), which can reach similar transformation efficiencies and level of complexity of foreign DNA insertion (Jackson et al., 2013; Wu et al., 2015) – the nature of complexity refers to positional and copy number of transgene insertion, which promotes a critical condition for silencing and stability of the transgene (Matzke et al., 1994; Iglesias et al., 1997; Waterhouse et al., 2001). Finally, foreign DNA insertion and successful expression of the transgene can be less critical than other factors with higher complexity, such as sugarcane genome size and polyploidy, which comprise a real challenge in this crop.

Despite the progress on transformation methodologies in sugarcane (**Figure 5**), transgene-free genome editing often requires the delivery of RNP complexes or transient expression systems into protoplasts (González et al., 2021; Lin et al., 2022; Sidorov et al., 2022). However, obtaining regenerated sugarcane plants from protoplasts is indeed a herculean task (Taylor et al., 1992), thus demanding efficient protocols for sugarcane protoplast regeneration (Hussin et al., 2022). Alternatively, delivery of CRISPR reagents directly into plant cells can be conducted via particle bombardment of embryogenic cells and zygotes, as reported in maize (Svitashev et al., 2016), wheat (Liang et al., 2017), and rice (Toda et al., 2019). Since their polyploidy and heterozygous nature hinder the use of sugarcane zygotes as targets for bombardment, a possible alternative is the bombardment of CRISPR reagents on somatic embryogenic cells or somatic embryos.

A significant concern in the genetic transformation of *Saccharum* genotypes lies in genotype-dependent responses to *in vitro* tissue culture procedures (**Figure 5**) (Di Pauli et al., 2021; Li et al., 2021). Several elite cultivars are recalcitrant to genetic transformation, thus hindering even conventional transgenesis (Altpeter et al., 2016). Although there are a few dozen genetically engineered *Saccharum* genotypes in the literature, e.g., ROC22 (China) (Wang et al., 2017), RB855156 and SP80-3280 (Brazil) (Reis et al., 2014; Cristofoletti et al., 2018), Co 86032 (India) (Augustine et al., 2015) and Q117 (Australia) (Jackson et al., 2013), there are many varieties of commercial interest around the world (Cursi et al., 2022; Zhao et al., 2022). To illustrate, our team witnessed energy cane genotypes (*S. spontaneum* x *S.* spp. hybrids) that are recalcitrant for callus production, whereas others produce heterogeneous calluses (embryogenic and non-embryogenic) with low regeneration efficiency (unpublished data). Moreover, some calluses regenerate but are incompatible with *Agrobacterium*-mediated transformation, for which bombardment transformation is encouraged. As a prospect, a versatile platform for simultaneous genome editing and transcription activation of morphogenetic regulators (e.g., *BABY BOOM*, *WUSCHEL2*) - CRISPR-Combo (Pan et al., 2022) - would be an innovative strategy to circumvent such plant regeneration barriers as well as to ease the screening of transgene-free genome-edited crops, as reported in rice plants. However, the search for tissue-specific promoters for sugarcane is warranted since these morphogenetic regulators may lead to phenotypic abnormalities and plant sterility under the activity of strong constitutive promoters (Lowe et al., 2018).

Chimerism and mosaicism are other unwanted phenomena derived from tissue culture. In order to avoid this, it is mandatory to regenerate an organism from a single genetically modified cell. The critical concerns for this are: (i) an efficient genetically modified cell selection system in the initial phase of tissue culture; and (ii) regeneration via somatic embryogenesis instead of organogenesis (Dong and McHughen, 1993). In transgenic sugarcane, the high-efficiency selection markers are the geneticin-G418 antibiotic (selectable marker gene: *nptII*) (Bower et al., 1996) and glufosinate herbicide (selectable marker gene: *bar*) (Enríquez-Obregón et al., 1998). Additionally, the promoters regulating selectable genes must also be efficient to obtain low transgene-copy plants, as opposed to low-ploidy plants, in which the selectable gene promoter is typical of low expression. Particularly for sugarcane, the use of strong promoters is recommended, such as *Zea mays* ubiquitin (pZmUbi) (Luo et al., 2022) and the enhanced CaMV 35S promoter (Kim et al., 2017; Guidelli et al., 2018).

Nevertheless, sugarcane tissue culture is time-consuming and plagued with high costs, thus demanding efforts for the application of viral vector-mediated CRISPR delivery. Viral vectors have been frantically tested and prospected towards the delivery of CRISPR machinery into plant cells (Oh et al., 2021); yet, there are no successful reports on sugarcane. Considering the biological nature of these systems, as *Agrobacterium*-mediated transformation, they rely on compatible biological interactions with the explant. In wheat, the *Barley stripe mosaic virus*-based sgRNA delivery vector (BSMV-sg) is effective in performing heritable genome editing (Li et al., 2021; Wang et al., 2022), thus raising potential applications in other monocot plants. The recombinant *Sugarcane mosaic virus* (SCMV) (Beernink et al., 2021) and the *Foxtail mosaic virus* (FoMV) (Mei et al., 2019) may also be other viable VIGE alternatives for testing in *Saccharum* spp. plants. Nevertheless, these viral vector-based approaches do not support the efficient delivery of large Cas effectors (more than 1000 amino acids), such as Cas9 (Baltes et al., 2014; Uranga et al., 2021), thus relying on Cas9-overexpressing plants (Li et al., 2021). However, small Cas effectors have been unveiled as efficient tools for VIGE in plants, highlighting the miniature Cas12f (Wu et al., 2021) and the hypercompact Cas12j2 (Liu et al., 2022) as promising candidates. As a prospect, this strategy may promote the accelerated genome editing of recalcitrant sugarcane cultivars in a high-throughput manner.

Finally, the highly-polyploidy nature of the sugarcane genome often impairs the screening of gene-edited events by conventional methods, such as T7 endonuclease 1 (T7E1) (Li et al., 2013), Surveyor nuclease (Cong et al., 2013), high-resolution melting (HRM) analysis (Montgomery et al., 2007) and direct sequencing of PCR-amplified target regions (Brinkman et al., 2014). Alternatively, the preliminary detection of sugarcane mutants by PCR/RE or capillary electrophoresis (Jung and Altpeter, 2016) is feasible but relies on robust validation approaches, such as pyrosequencing (Jung and Altpeter, 2016). Moreover, novel high-throughput and low-cost genotyping methods, such as HI-TOM (Liu et al., 2019) and CRIS.py (Connelly and Pruett-Miller, 2019), are critically relevant for the massive screening of marker-free, genome-edited sugarcane plants.

To date, genome editing technologies are still in their infancy in sugarcane biotechnology (**Table 4**). However, Altpeter and his colleagues have pioneered elementary molecular strategies employing TALEN and CRISPR/Cas9 systems in these crops (Jung and Altpeter, 2016; Kannan et al., 2018; Eid et al., 2021; Oz et al., 2021). Firstly, they implemented TALEN technology for multiallelic mutagenesis of a lignin biosynthetic gene, *caffeic acid O-methyltransferase* (*COMT*), which improved the saccharification efficiency (54%) without impairing biomass yield (Jung and Altpeter, 2016; Kannan et al., 2018). Further on, Altpeter’s team unlocked the application of the CRISPR-Cas9 system in sugarcane. As a proof of concept, a highly evident phenotype was elicited by multiallelic, targeted mutagenesis of *magnesium chelatase subunit I* (*MGCH*), a gene encoding a key enzyme for chlorophyll biosynthesis. Furthermore, in this study, they performed a heat treatment of transformed sugarcane calli, which increased the gene editing frequency by 2-fold and enabled the visual identification of the yellow leaf color phenotype (Eid et al., 2021). Strikingly, in another study, a co-editing of multiple alleles was carried out on the *acetolactate synthase* (*ALS*) gene involving two amino acid substitutions (W574L and S653I) inserted by template-mediated HDR (Oz et al., 2021). Among the strains that simultaneously bore the W574L and S653I substitutions, the acquired herbicide (nicosulfuron) resistance was displayed in the entire foliage (Oz et al., 2021).

**Table 4:** Summary of reports on sugarcane genome editing and details concerning genetic engineering and CRISPR/Cas parameters.

Technique	Transformation method	Strategy	Delivery	Mutation rate	Target gene	Desired trait	Reference
TALEN	*A. tumefaciens*-mediated	KO	Plasmid DNA	74%	*COMT*	Reduced lignin content and increased saccharification efficiency	Jung and Altpeter (2016)
	Biolistics		Expression cassette	30%			
TALEN	*A. tumefaciens*-mediated	KO	Plasmid DNA	up to 89.3%	*COMT*	Reduced lignin content and increased saccharification efficiency	Kannan et al. (2018)
	Biolistics		Expression cassette	up to 92.5%			
CRISPR/Cas9	Biolistics	KO	Expression cassette	up to 83.1%	*MGCH*	Scorable phenotype (yellow leaf color)	Eid et al. (2021)
CRISPR/Cas9	Biolistics	KI	Expression cassette	up to 11.6%	*ALS*	Herbicide resistance	Oz et al. (2021)
CRISPR/Cas9	Biolistics	KO	Ribonucleoprotein	0.01%	*BAHD01*	Enhanced biomass saccharification	Unpublished
					*BAHD05*	Increased sugar concentration	

In 2022, a massive team of the Brazilian Agricultural Research Corporation (EMBRAPA), was able to deploy a laborious strategy via biolistics by using microparticles carrying RNPs and bombarding onto sugarcane embryogenic calli. They knocked out two genes, *BAHD01* and *BAHD05*, which resulted in increased biomass saccharification and sugarcane concentration, respectively (unpublished data). Although this marker-free selection approach relies on a time-consuming screening of rare gene-edited plants, it can ease the biosafety deregulation process to launch a biotechnological product on the market in some countries, as mentioned before. Collectively, these studies have established early strategies for optimizing sugarcane genome editing, thereby overcoming some hurdles of a highly polyploid genome. Nevertheless, whilst other sophisticated CRISPR systems (e.g., base editing and prime editing) have already been employed in several plant crops (Bharat et al., 2020; Molla et al., 2021), CRISPR-based sugarcane genome editing is still restricted to traditional mutagenesis with Cas9 nucleases (Eid et al., 2021; Oz et al., 2021). Therefore, proof-of-principle studies regarding the usage of other nucleases and robust base- and prime-editing systems in sugarcane are still warranted.

## 7 Global regulatory scenario for edited plants

In addition to functional genomics, the commercialization of genome-edited plants is a highly sought-after goal. For almost 30 years, several countries have released transgenic cultivars for commercial planting. So far, 439 events of 32 cultivars of transgenic plants were approved for commercialization in 45 countries, according to the GM approval database from the International Service for the Acquisition of Agri-Biotech Applications (ISAAA, 2022). Most commercialized transgenic plants meet characteristics of interest to the farmer, such as herbicide tolerance and insect resistance (ISAAA, 2018). However, although genetically modified organisms (GMOs) have been on the market for a long time to prove their safety, they still encounter some barriers in society, such as concerns about allergenicity, adverse effects on the environment, and even issues involving intellectual property and hegemony of large companies (Bawa and Anilakumar, 2013; Caserta and de Souza, 2017).

Commercial release of these plants for cultivation obeys careful legislation and different legal frameworks worldwide. Generally, the requirements for releasing a transgenic plant are only sometimes proportional to the risks involved. In other words, regulatory obstacles can be so severe that only large corporations can finance this long process. The legislation of different countries may be oriented toward analyses that consider the process or the product (Turnbull et al., 2021; Ahmad et al., 2023).

The advent of genome editing expanded the possibilities within plant biotechnology, especially due to CRISPR/Cas technology. Since then, countries have been forced to rethink whether their legislation aimed at transgenic plants includes the new possibilities of this toolbox. This demand is because, through genome editing, plants can be generated with precise mutations that could have occurred naturally by spontaneous mutations or even by sexual crossing between compatible species (Custers and Dima, 2022). Moreover, directed mutagenesis and classical breeding, outside the scope of legislation involving transgenic plants, can generate much more significant effects on the genome of plants than genome editing (Ossowski et al., 2010).

**Figure 6:**
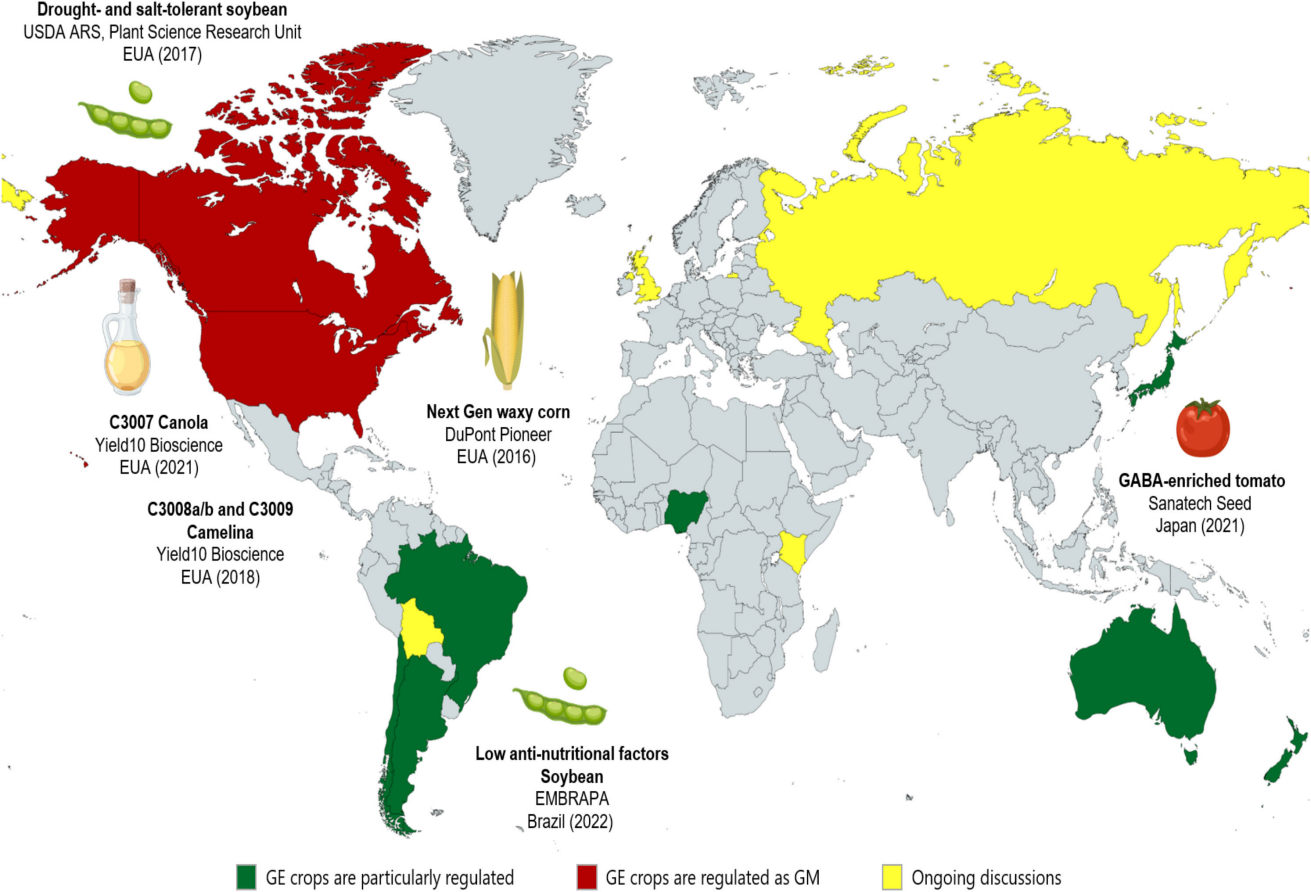
Current status of global legislation on plant genome editing. Genome-edited (GE) crops are distinctively (particularly) regulated worldwide, as illustrated. Green areas represent countries that address GE and genetically modified (GM) crops with different standards, in which most SDN-1 and SDN-2 are granted a free pass. Although some countries maintain similar regulation between GE and GM crops (in red), these include the USA, which has the largest number of CRISPR-based products released. Other CRISPR-based products have also been released in Japan and Brazil. Meanwhile, the yellow-colored areas represent countries that are still reviewing their deregulation rules for GE crops. Figure elements were obtained from BioRender.com.

The unique characteristics of each gene-edited plant led many countries to elaborate on specific legislation that recommends a case-by-case analysis, as explained below. Additionally, to support their legislation, many countries adopted the definition of Living Modified Organisms (LMOs) included in the Cartagena Protocol on Biosafety of the Convention on Biological Diversity. Thus, some countries interpret that, concerning edited organisms, only organisms that have a modification that results in a new combination of genetic material and would not occur naturally can be treated as LMOs (Whelan and Lema, 2015). This possibility does not classify as LMOs the cases of site-directed nucleases (SDN)-1 and some cases of SDN-2 (Rostoks, 2021). From this, non-LMO organisms can be regulated without following the rules of transgenic plants. Additionally, crops can be distinguished between genetically modified (GM) and genome-edited (GE) plants according to a case-by-case analysis from what each country considers to be or not a GE crop and, in this way, information about some countries and their respective legislation concerning GM and GE crops is summarized in **Table 5** and **Figure 6** A comprehensive source on the subject, mainly on GE plants, was described by Molinari et al. (2021).

**Table 5:** Comparison of regulatory distinctions between Genetically Modified (GM) and Genome-Edited (GE) plants.

Country	Legislation about commercial GM release	GM plant regulatory approaches	Legislation about commercial GE release	GE plant regulatory approaches	References
Brazil	Liberal	Process	Different of GM	Case-by-case	(Ishii and Araki, 2017; El-Mounadi et al., 2020; Kuiken and Kuzma, 2021)
Argentina	Liberal	Product	Different of GM	Case-by-case	(El-Mounadi et al., 2020; Kuiken and Kuzma, 2021)
Bolivia	Liberal	Process	–	In review	(Kuiken and Kuzma, 2021)
Chile	Prohibitive	–	Different of GM	Case-by-case	(Kuiken and Kuzma, 2021; Rosado and Eriksson, 2022)
Venezuela	Prohibitive	–	–	Undefined	(Gatica-Arias, 2020)
Ecuador	Prohibitive	–	–	Undefined	(Gatica-Arias, 2020; Rosado and Eriksson, 2022)
United States of America	Liberal	Product	Same of GM	Case-by-case	(Entine et al., 2021; Jenkins et al., 2021)
Canada	Liberal	Product	Same of GM	Case-by-case	(Rowand, 2009; Smyth, 2017; Jenkins et al., 2021)
South Africa	Liberal	Process	–	Undefined	(Turnbull et al., 2021; Rozas et al., 2022; Sprink et al., 2022)
Nigeria	Liberal	Process	Different of GM	Case-by-case	(Jenkins et al., 2021; Rozas et al., 2022; Sprink et al., 2022)
Kenya	Liberal	Product	–	Case-by-case	(Turnbull et al., 2021; Rozas et al., 2022; Sprink et al., 2022)
Egypt	Prohibitive	–	–	Undefined	(Turnbull et al., 2021)
Ghana	Prohibitive	–	–	Undefined	(Turnbull et al., 2021)
Uganda	Prohibitive	–	–	Undefined	(Turnbull et al., 2021)
European Union (except Portugal and Spain)	Prohibitive	–	–	Undefined	(Custers and Dima, 2022)
Portugal	Liberal	Product	–	Undefined	
Spain	Liberal	Product	–	Undefined	
England	Prohibitive	–	–	In review	(Custers and Dima, 2022)
Russia	Prohibitive	–	–	In review	(Bogatyreva et al., 2021)
Philippines	Liberal	Product	–	Undefined	(Bogatyreva et al., 2021; Sprink et al., 2022)
Bangladesh	Liberal	Product	–	Undefined	(Bogatyreva et al., 2021; Rozas et al., 2022)
India	Liberal	Process	–	Prohibitive	(El-Mounadi et al., 2020; Sprink et al., 2022)
China	Liberal	Process	–	In review	(Rozas et al., 2022; Sprink et al., 2022)
Japan	Only ornamental plants	–	Different of GM	Case-by-case	(Ishii and Araki, 2017; Entine et al., 2021; Nagamine and Ezura, 2022; Rozas et al., 2022; Sprink et al., 2022)
Korean Republic	Prohibitive	–	–	Undefined	(Ishii and Araki, 2017; Sprink et al., 2022)
Australia	Liberal	Process	Different of GM	Case-by-case	(Thygesen, 2019; Entine et al., 2021)
New Zealand	Prohibitive	–	Different of GM	Case-by-case	(Ishii and Araki, 2017; Thygesen, 2019; Entine et al., 2021)

Soon after the first cases of genome editing in plants, in 2013, Argentina was the pioneer in establishing specific legislation for edited plants, opting for the case-by-case approach (Whelan and Lema, 2015). Despite not being a signatory country of the Cartagena Protocol, Argentina relied on the definition of LMOs to define its regulatory strategies. Argentinian legislation particularly includes the possibility of previous analyses of the publishing project through the Prior Consultation Instance (PCI) (Goberna et al., 2022). Through these early analyses, the developer can indicate which legislation he must comply with to regulate his product (plant, microorganism or animal). Even so, if the PCI analysis indicates that the product is not characterized as an LMOs, a new consultation is necessary after finalizing the project (Goberna et al., 2022). Due to the possibility of prior consultation, Argentina has generated an increased interest in gene editing by developers.

Biotechnology has evolved rapidly with transgenic plants. However, the most significant breakthrough took place a decade ago, with the discovery of genome editing through the CRISPR/Cas system (Jinek et al., 2012; Gostimskaya, 2022). Since then, worldwide regulatory guidelines are no longer valid to comprehend the plants that originated with this technology. In this way, many countries updated their legislation quickly in search of greater socioeconomic advances. However, as explained above, although often indistinguishable from natural mutations, the mutations caused by genomic editing still face legal barriers and acceptance in some countries. For instance, despite the ultra-restrictive GMO regulation, the European Commission has recently proposed loosening the rules for GE crops to treat them as conventionally bred (Stokstad, 2023). This could not only foster scientific research into GE plants but also accelerate the launch of sustainable products onto the market. However, there are still conservationist counterforces slowing down this progress.

Despite this, founded on analyzes based on science and bioeconomy, several countries already have specific legislation to deal with these cases (Ahmad et al., 2023). In a general context, it is clear that the deregulation of SDN-1 and, in some cases, of SDN-2 in particular countries seeks to meet different purposes and results in different benefits. The Argentinean example of the possibility of prior consultation regarding the deregulation of some edited plants makes it possible to see some of these advantages immediately. While GMOs are mostly restricted to a few large corporations, one of the apparent consequences of the Argentine guidelines on edited plants is the greater interest in investing in this sector by small and medium-sized private companies or public research sectors from the country (Whelan et al., 2020). The increased interest in smaller companies is due to the decrease in the time to produce these plants and the costs of deregulation (Lassoued et al., 2019). Thus, it is common sense that CRISPR/Cas technology democratized plant genetic engineering, making it widely accessible to researchers and companies and making engineered plants accessible to growers and consumers. In addition, the new laws introduced by the regulatory structures of different countries are concerned with facilitating trade, improving the economy and contributing to food security.

Finally, it is important to consider that GE crops acceptance will also depend on clarification of the precision level of genome editing process, by means of assessing unintended or unpredictable off-target edits. In this way, interpreting genome-wide effects should be aligned with comparative evaluation of the degree of mutations with crops developed through traditional breeding. Thus, strategies directed towards avoidance of unintended changes (e.g., ensuring accuracy of gene editing through optimization of on-target activity) can help to clarify regulatory agencies about the possibility to circumvent off-target effects and, consequently, reduce concerns of risks involving GE crops (Zhao and Wolt, 2017). Moreover, differently from human health products derived from genome editing, off-target mutations and deleterious effects in plants tend to be less critical and pose no ethical issues (Schmidt et al., 2020), thus possibly reducing public concern and circumventing regulatory barriers limiting market approval.

## 8 Perspectives and challenges

CRISPR/Cas technology has revolutionized genetic engineering by enabling precise and efficient modification of DNA sequences in many annual plants. However, its application in perennial/semi-perennial plants presents challenges and promising prospects that deserve thorough exploration.

Unlike annuals, perennial plants have extended life cycles and slower reproduction rates. This characteristic hinders the development of CRISPR-edited plants with desired mutations and their phenotypes, since multiple growing seasons are required to observe the intended phenotypic changes. In addition, perennials interact with their environment differently from annuals, potentially impacting the expression of modified genes. Understanding the intricate relationships between perennial plants and their surroundings is essential to predict the environmental effects of CRISPR modifications. Moreover, CRISPR/Cas systems can introduce unintended genetic alterations, raising concerns about the potential off-target effects that might accumulate over the lifespan of perennial plants. Thus, ensuring accuracy and optimizing on-target activity is crucial to prevent unintended consequences. On the other hand, once the phenotypic changes are characterized, the selected modified plants can be used to insert other genes in the same location. This process avoids several years of selection for events in which the gene may have been inserted in undesirable locations in the genome, as occurs in traditional transgenesis approaches. Therefore, it is expected that, in the future, obtaining edited perennial plants with desirable characteristics will be a less laborious and faster technology to be achieved.

Overall, CRISPR can be employed to improve fruit quality, increase disease resistance, and increase the tolerance of perennial crops to abiotic stresses. These modifications could have significant positive implications for food security and agricultural sustainability. Perennial plants often face several environmental stresses over their long lifespans, and CRISPR technology offers the potential to enhance their adaptability to climate changes and other environmental challenges, making them more resilient. Hence, overcoming the challenges of applying CRISPR to perennial plants will lead to significant scientific insights into plant genetics, gene regulation, and long-term evolutionary processes. In conclusion, the use of CRISPR in perennial plants shows great potential to revolutionize agriculture and contribute to food security. While challenges related to long reproductive cycles, off-target effects, and regulatory considerations exist, the prospects of enhancing fruit yield and stress tolerance make this technology highly promising. As scientific researchers address these challenges, they pave the way for a more sustainable and resilient agricultural future.

## Author contributions

GP: Conceptualization, Writing – original draft, Writing – review & editing. DR: Writing – original draft, Writing – review & editing. LS: Writing – original draft, Writing – review & editing. DC: Writing – original draft, Writing – review & editing. PN: Writing – original draft. JS: Writing – original draft. LP: Writing – original draft. MM: Writing – original draft, Writing – review & editing, Funding acquisition, Supervision. GL: Writing – original draft, Writing – review & editing. TP: Writing – review & editing. CV: Writing – original draft. SC: Funding acquisition, Supervision, Writing – review & editing. RB-C: Writing – review & editing. MT: Writing – review & editing. MY: Funding acquisition, Resources, Supervision, Writing – review & editing. AS: Conceptualization, Funding acquisition, Resources, Supervision, Writing – original draft, Writing – review & editing.
